# Machine learning of charges and long-range interactions from energies and forces

**DOI:** 10.1038/s41467-025-63852-x

**Published:** 2025-10-01

**Authors:** Daniel S. King, Dongjin Kim, Peichen Zhong, Bingqing Cheng

**Affiliations:** 1 Bakar Institute of Digital Materials for the Planet, UC Berkeley, California, CA USA; 2 Department of Chemistry, UC Berkeley, California, CA USA; 3 The Institute of Science and Technology Austria, Am Campus 1, 3400 Klosterneuburg, Austria

**Keywords:** Computational science, Method development, Atomistic models

## Abstract

Accurate modeling of long-range forces is critical in atomistic simulations, as they play a central role in determining the properties of material and chemical systems. However, standard machine learning interatomic potentials (MLIPs) often rely on short-range approximations, limiting their applicability to systems with significant electrostatics and dispersion forces. We recently introduced the Latent Ewald Summation (LES) method, which captures long-range electrostatics without explicitly learning atomic charges or charge equilibration. We benchmark LES on diverse and challenging systems, including charged molecules, ionic liquids, electrolyte solutions, polar dipeptides, surface adsorption, electrolyte/solid interfaces, and solid-solid interfaces. Here we show that LES can reproduce the exact atomic charges for classical systems with fixed charges and can infer dipole and quadrupole moments, as well as the dipole derivative with respect to atomic positions, for quantum mechanical systems. Moreover, LES can achieve better accuracy in energy and force predictions compared to methods that explicitly learn from charges.

## Abstract (web summary)

Long-range interactions are challenging for machine learning interatomic potentials (MLIPs). Here, authors show that, by just learning from energies and forces, MLIPs can accurately capture electrostatics and predict atomic charges.

## Introduction

The accurate incorporation of long-range interactions in atomistic simulations of materials and chemical systems remains a fundamental challenge^1^. Early approaches to address this issue included the cluster expansion formalism for crystalline lattices^2^, parameterization of classical force fields with fixed charges^3^, and charge equilibration schemes^4^, among others.

The proliferation of machine learning interatomic potentials (MLIPs)^5,6^, which learn surrogate potential energy surfaces from quantum mechanical reference calculations of atomic configurations, has further emphasized the need for accurately accounting for long-range interactions. Most established MLIP frameworks rely on short-range approximations, assuming that the energy contribution of each atom is determined by its local atomic environment. While this assumption enables computationally efficient linear scaling with respect to system size, it poses significant limitations for systems where long-range interactions, such as electrostatics, play a critical role. These limitations are particularly evident in systems involving electrochemical interfaces^7^, charged molecular dimers^8,9^, ionic^10^ and polar materials^11^, and scenarios involving varying charge states or long-range charge transfer^12^.

One option is to predict effective partial charges of each atom, which are then used to determine long-range electrostatics^12–17^. For example, the third-generation HDNNP (3G-HDNNP)^12^ contains electrostatic interactions based on local environment-dependent charges represented by atomic neural networks. To improve upon that, the fourth-generation high-dimensional neural network potentials (4G-HDNNPs)^12^ predict the electronegativities of each atom and then use a charge equilibration scheme^18^ to assign the charges. 3G-HDNNPs and 4G-HDNNPs are trained directly to reproduce atomic partial charges from reference quantum mechanical calculations, although partial charges are not physically observable and their values depend on the specific partitioning scheme used^15^. Another approach is to learn the maximally localized Wannier centers (MLWCs) for insulating systems: the deep potential long-range (DPLR) model^10^ computes the long-range electrostatics using spherical Gaussian charges associated with the nuclei and the average positions of the MLWCs predicted via a Deep Wannier (DW) deep neural network model based on the local chemical environment^10^. The charges of these MLWCs are based on the number of valence electrons of each element. A similar method is the self-consistent field neural network (SCFNN)^14^, which predicts the electronic response via the position of the MLWCs.

There are a few other methods that do not explicitly learn the atomic charges^8,9,11,19–21^. For example, the Ewald message-passing method^20^ employs a learnable frequency filter in the reciprocal space to generate a long-range message for each atom during the message-passing step. RANGE^22^ creates global virtual nodes in message-passing graph networks to aggregate and broadcast long-range information. LODE^8,9,23^ computes the potential field generated by all the atoms in the system in the reciprocal space via Ewald summation, and then featurizes such field near a central atom up to some cutoff radius to form the long-range descriptors. The density-based long-range descriptor^21^ follows a similar procedure, but the global atomic density itself is used instead of the field.

Recently, we introduced the Latent Ewald Summation (LES) method^24^. LES decomposes the total potential energy into short-range and long-range components. Hidden variables–interpreted as latent charges–are predicted from local atomic features without reference to specific charge definitions. These latent charges are then used to predict the long-range potential via an Ewald summation. LES can be combined with any short-ranged MLIP architectures (e.g., HDNNP^25^, Gaussian Approximation Potentials (GAP)^26^, Moment Tensor Potentials (MTPs)^27^, atomic cluster expansion (ACE)^28^) and message passing neural networks (MPNNs) (e.g., NequIP^29^, MACE^30^). We combine LES with Cartesian atomic cluster expansion (CACE)^31^, and refer to the standard short-ranged CACE as CACE-SR, and the combined long-range potential as CACE-LR.

In this paper, we provide a comprehensive exploration of the LES framework, detailing its theoretical foundation, possible extensions, and application to a range of test systems. Importantly, we show that, when limited to a single charge channel, the LES charges can be interpreted as physical partial charges. In ref. ^24^, LES was compared to other LR methods such as LODE^8,9^ and density-based long-range descriptor^21^ that do not explicitly learn charges. Here, we further compare LES to existing methods that incorporate long-range interactions via explicit charge learning and show that LES achieves superior performance.

## Results

### Theory

We first briefly recap LES^24^, and then make an explicit connection between LES and physical charges. Finally, we briefly demonstrate how different global charge states can be encoded in the LES framework.

#### Range separation

The total potential energy of a system with *N* atoms is split into short-range (SR) and long-range (LR) components, $E={\sum }_{i=1}^{N}{E}^{{{\rm{sr}}}}({B}_{i})+{E}^{{{\rm{lr}}}}$. The short-range energy is the sum of atomic energies, each depending on local *B* features of atom *i*. The *B* features can be local atomic environment descriptors such as ACE^28^,

or learned features in message passing neural networks (MPNNs)^29,32–34^. For the long-range part, a multilayer perceptron with parameters *ϕ* maps the invariant features of each atom *i* to a hidden variable:

1 $${q}_{i}={Q}_{\phi }({B}_{i}).$$

In general, *q* can be multi-dimensional to represent the generalized long-range interactions. When *q* is restricted to be one-dimensional, it can be interpreted as the atomic charge as we discuss later.

Suppose that the potential-generating field by a single particle with unity latent variable is proportional to *u*(**r**) = ∣**r**∣^−*p*^, with *p* being a fixed exponent. Following the standard range-separation formalism^35^, one can express short-range and long-range interactions by multiplying the interaction by a convergence function *φ*(*r*) with *φ*(0) = 1 decreasing rapidly to zero as *r* increases:

2 $${E}_{p}=\,	{E}_{p}^{{{\rm{sr}}}}-{E}_{p}^{{{\rm{self}}}}+{E}_{p}^{{{\rm{lr}}}} \\=	\frac{1}{2}\mathop{\sum }_{i\ne j}{q}_{i}{q}_{j}{r}_{ij}^{-p}\varphi ({r}_{ij})-\frac{1}{2}\sum _{i=1}^{N}{q}_{i}^{2}{\lim }_{r\to 0}{r}^{-p}[1-\varphi (r)]\\+	 \frac{1}{2}\sum _{i=1}^{N}\sum _{j=1}^{N}{q}_{i}{q}_{j}{r}_{ij}^{-p}[1-\varphi ({r}_{ij})].$$

Both ${E}_{p}^{{{\rm{sr}}}}$ and ${E}_{p}^{{{\rm{self}}}}$ are short-ranged in nature and can be described by the short-ranged MLIP based on the local features.

#### Long-range energy

For *p* = 1, which corresponds to electrostatics, one choice for the convergence function can be expressed as the complementary error function $\varphi (r)={{\rm{erfc}}}(\frac{r}{\sqrt{2}\sigma })$. For isolated systems without periodic boundary conditions, one can compute the ${E}_{p}^{{{\rm{lr}}}}$ term directly in the real space based on enumerating pairwise distances between atoms. For periodic systems, the corresponding long-range electrostatics can be computed in the reciprocal space as

3 $${E}_{1}^{{{\rm{lr}}}}=\frac{2\pi }{V}\sum_{0 < k < {k}_{c}}\frac{1}{{k}^{2}}{e}^{-{\sigma }^{2}{k}^{2}/2}| S({{\bf{k}}}){| }^{2},$$

where the structure factor *S*(**k**) of the hidden variable is defined as

4 $$S({{\bf{k}}})=\sum_{i=1}^{N}{q}_{i}{e}^{i{{\bf{k}}}\cdot {{{\bf{r}}}}_{i}}.$$

The omission of the *k* = 0 term in Eq. (3) means the tinfoil boundary condition is applied. The detailed derivations and the case for *p* = 6 which corresponds to London dispersions can be found in the Supplementary Information.

#### Learning charges from energy and forces

When training the MLIP, the total potential energy *E*, interatomic forces **F**_*i*_ = −∂*E*/∂**r**_*i*_, and sometimes virial stress are fitted to the reference values from the dataset. In LES, unlike methods that explicitly learn partial charges, the hidden variables *q* are hypothesized to represent flexible atomic charges when the physical electrostatic constant 1/4*πϵ*_0_ is included. In particular, when LES is limited to a single charge channel, we find that the charge used to compute the long-range energy in Eq. (3) is physically meaningful and can be used to predict physical observables such as the dipole moment of gas phase molecules and Born effective charges^36^. However, it is noted that because the structure factor is squared in Eq. (3), the predicted charges do not distinguish the charge parity, as the total energy stays the same if all signs of the charges are flipped. In practice, it is easy to unflip the signs of atomic charges based on the known electronegativity of elements.

We note the success of the LES method in predicting charge locally while computing energy globally. This choice reflects the generally nearsightedness of electron matter^37^. Additionally, as LES learns the charges via the energy and forces, its learning is flexible to arbitrary charge distributions (e.g., different oxidation states) as long as they have an impact on the energy in the training set. Indeed, the LES approach proves appropriate for a wide range of systems such as electrolyte/electrodes, charged molecules, and doped surfaces, as we will show in the examples. However, it is important to note that while the local charge assumption works well empirically, it lacks theoretical guarantees and may encounter limitations in specific edge cases, such as systems involving long-range charge transfer.

#### Charge neutrality condition

Empirically, in the examples below and in previous work^24^ we have found it unnecessary to explicitly enforce charge neutrality or fixed total charge state in the training process of LES. In practice, we have found that the sum of *q* is usually close to the total charges for both neutral and charged systems without enforcing neutrality. Additionally, any residual difference is treated as a uniform background charge, which does not affect the total energy as the *k* = 0 term is omitted in the reciprocal space computation of electrostatic interactions in Eq. (3). In all the examples we have tested, we did not observe any loss of accuracy or artifacts due to the lack of charge equilibration. In contrast, for the ML models that explicitly learn charges such as 3G-HDNNP^12^, the lack of charge equilibration may result in dramatically larger errors, and sometimes pathological behaviors were observed for systems involving charge transfer and change of charge states.

#### Different charge states

In a standard MLIP, the atomic features *B*_*i*_ depend on the chemical elements and the coordinates of the atoms surrounding atom *i*, and are agnostic to the charge or oxidation state. This means that two systems with identical atomic positions but different net charges *Q* will have degenerate features. Although this degeneracy does not affect the training or prediction for systems with a fixed net charge, it can cause problems when handling systems with varying charge states simultaneously. To resolve this, in training sets containing multiple net charges (only one of the examples below, ${{{\rm{Ag}}}}_{3}^{+}/{{{\rm{Ag}}}}_{3}^{-}$) we concatenate the total charge *Q* of the system with the local atomic features *B*_*i*_, *B*_*i*_ ⊕ *Q*, and use this combined feature as the input for predicting short-range atomic energies and local hidden variables. Note that this global charge embedding scheme can have limitations, for example, when dealing with charged systems with varying sizes.

### Random charges

**Fig. 1:**
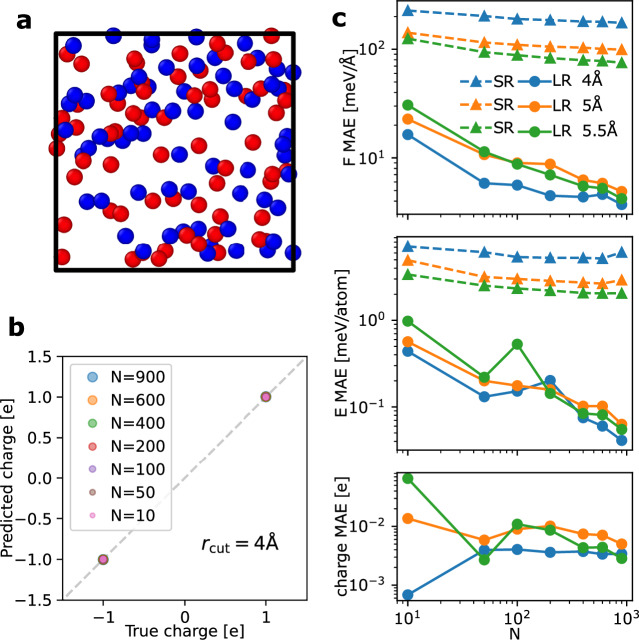
Benchmark of the short-range and long-range models on the system of point charges. **a** A configuration of gas made of point charges. The red and the blue colors refer to particles with +1e and -1e charges, respectively. **b** Comparison of the true and the predicted charges for the Cartesian atomic cluster expansion (CACE) long-range (CACE-LR) models with a cutoff radius *r*_cut_ of 4 Å and trained on *N* configurations. **c** The mean absolute errors (MAEs) on energy (E), forces (F), and charges for short-range (SR) and long-range (LR) models trained using different *N* numbers of samples.

As an initial test, a gas of point charges was constructed. As shown in Fig. 1a, each configuration consists of 128 atoms, with 64 carrying a positive charge of +1e and the remaining 64 carrying a negative charge of  −1e. The atoms interact through the Coulomb potential and the repulsive component of a Lennard-Jones potential. This benchmark aims to evaluate the learning efficiency of the LES framework and assess whether the correct atomic charges can be accurately learned. Unlike in density functional theory (DFT), where the precise values of partial charges depend on the chosen definition, the charges in this system are unambiguously defined.

For the short-range component, we employed CACE with different cutoff distances of *r*_cut_ = 4 Å, 5 Å, and 5.5 Å. For the long-range interactions, we used a one-dimensional *q* with *σ* = 1 Å in the Ewald summation, without enforcing a net charge constraint. Figure 1b presents the parity plot of the CACE-LR model with *r*_cut_ = 4 Å, comparing the true and predicted charges (after unflipping the charge parity) for various numbers of training samples. Remarkably, even with just 10 training configurations, the predicted charges are nearly exact.

Figure 1c illustrates the learning curves for the mean absolute errors (MAEs) in energy, forces, and charges, using short-range (SR) and long-range (LR) models with different cutoffs. The SR models exhibit slow learning and significant errors for this dataset, with performance improving as *r*_cut_ increases. In contrast, the LR models achieve errors more than an order of magnitude lower, with learning efficiency improving as *r*_cut_ decreases. This example highlights that, unlike the typical behavior of SR MLIPs, long-range potentials achieve more efficient learning with appropriately small *r*_cut_ values.

### Electrolyte solutions

**Fig. 2:**
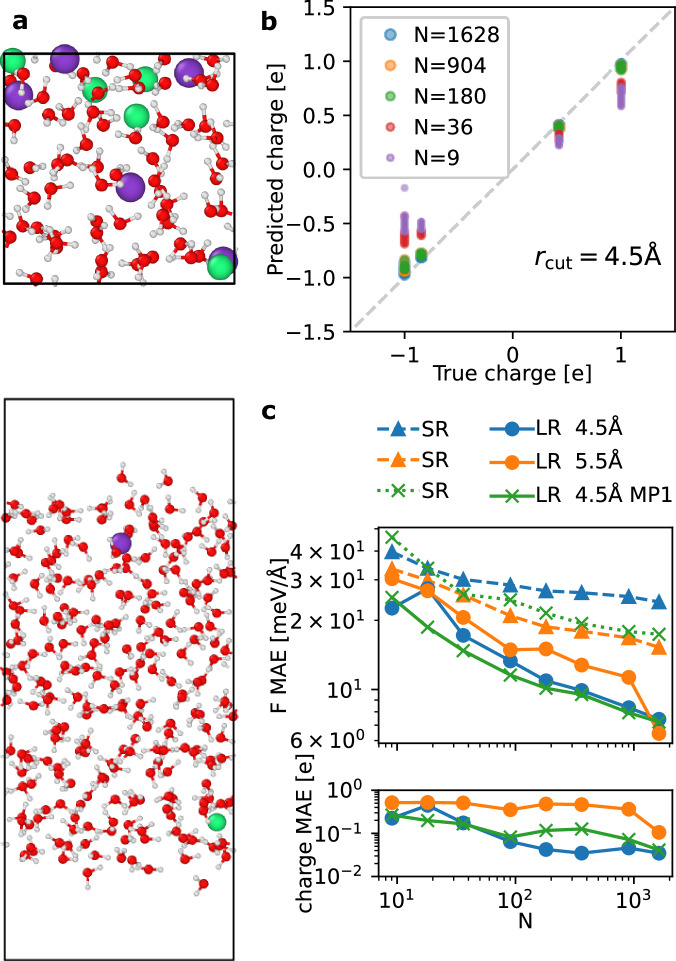
Benchmark of the short-range and long-range models on bulk and interfacial electrolytes. **a** A bulk electrolyte configuration (upper panel) and an electrolyte-vapor configuration (lower panel) of KF in water electrolyte of 1 KF ion pair and 233 water molecules, randomly selected from the training set. **b** Comparison of the true and the predicted charges for the Cartesian atomic cluster expansion (CACE) long-range (CACE-LR) models with a cutoff radius *r*_cut_ of 4.5 Å and trained on *N* configurations. **c** The mean absolute errors (MAEs) on forces (F), and charges for short-range (SR) and long-range (LR) models trained using different *N* numbers of samples. The MP1 indicates models using one message-passing layer.

We constructed a dataset of potassium fluoride (KF) aqueous solutions with concentrations ranging from 0 to approximately 2 mol/L. The dataset includes both bulk electrolyte solution configurations and electrolyte-vapor interfaces, as illustrated in Fig. 2. The reference energies and forces were computed using the flexible SPC/Fw water model (with oxygen carrying a charge of  −0.8476e and hydrogen carrying a charge of +0.4238e)^38^, alongside ions with fixed charges (K:  +1e, F:  −1e)^39^. It is worth noting, however, scaled charge ion models are typically better at capturing implicit effects of liquid-phase polarization and modeling electrolytes^3,40,41^, although this example aims to demonstrate the learning ability of MLIPs rather than to accurately model electrolytes. This electrolyte dataset is significantly more challenging than the random charge example, as it involves multiple species with distinct atomic charges. Additionally, water acts as a dielectric medium, and the presence of interfaces introduces diverse screening effects that vary with depth from the surface.

Figure 2 b shows that the CACE-LR model with *r*_cut_ = 4.5 Å is able to recover the true charges after a couple of hundred training samples. Figure 2c shows the learning curves for the MAEs on forces and charges, and the MAEs on energies are all pretty small for all models (< 0.3 meV/atom for ⪆ 100 samples). While a larger cutoff or a message passing layer (MP1) improves the SR model, the LR model with a smaller cutoff *r*_cut_ = 4.5 Å achieves better learning efficiency. Adding a message-passing layer to the LR model has little effect in this case. See below in the Methods section, we also show the learning curves from just the bulk or just the interfacial configurations. This electrolyte example shows that the LR model is able to learn the charges and energetics of systems involving different species and a dielectric medium that screens electrostatics.

### Charged molecular dimers

**Fig. 3:**
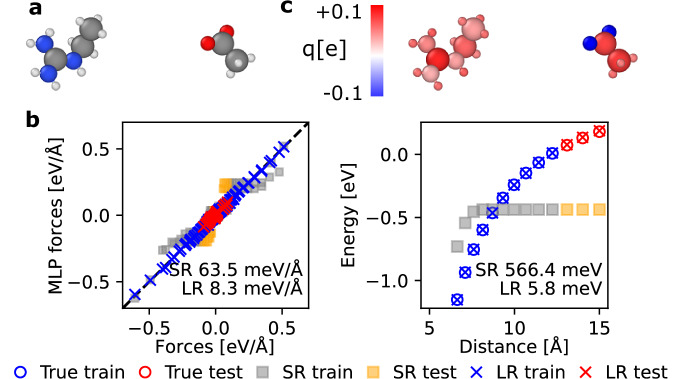
Benchmark of the short-range and long-range models on systems of molecular dimer. **a** A snapshot of the molecular dimer configuration of ${{{\rm{C}}}}_{3}{{{\rm{N}}}}_{3}{{{\rm{H}}}}_{10}^{+}$/${{{\rm{C}}}}_{2}{{{\rm{O}}}}_{2}{{{\rm{H}}}}_{3}^{-}$. **b** The comparison between the true and predicted force components (left panel), and the binding energy curves (the energy difference between the dimer and two isolated monomers) from short-range (SR) and long-range (LR) models. **c** The predicted charges *q* from the long-range model. The color bar is in linear scale.

We revisit an example from a molecular dimer dataset^42^ used to benchmark LODE^9^ and LES^24^. This example consists of the binding curve between two charged molecules of ${{{\rm{C}}}}_{3}{{{\rm{N}}}}_{3}{{{\rm{H}}}}_{10}^{+}/{{{\rm{C}}}}_{2}{{{\rm{O}}}}_{2}{{{\rm{H}}}}_{3}^{-}$ (shown in Fig. 3a). The training set of this example is tiny: it consists of 10 configurations of the dimer pair, with the internal coordinates of the molecules frozen and only dimer separation distances varying between ~5 Å and 12 Å. The test set includes 3 configurations with separations between approximately 12 Å and 15 Å. The dataset includes energy and force information calculated using the HSE06 hybrid DFT with a many-body dispersion correction.

For the CACE-LR model, here we use a one-dimensional *q*, whereas the original LES paper^24^ used a four-dimensional hidden variable, and the model test errors are comparable. Figure 3b compares the predicted forces and dispersion curves for the LR and SR models. The SR model has one message-passing layer, but as the two molecules can have a distance beyond the cutoff of *r*_cut_ = 5 Å, the message-passing scheme does not help. Figure 3c shows the predicted charge distribution. The total predicted charges on ${{{\rm{C}}}}_{3}{{{\rm{N}}}}_{3}{{{\rm{H}}}}_{10}^{+}/{{{\rm{C}}}}_{2}{{{\rm{O}}}}_{2}{{{\rm{H}}}}_{3}^{-}$ molecules are +0.83e/ −1.08e, and  +1.01 e/ −1.01 e after removing the mean charge of each atom ${q}_{i}\leftarrow {q}_{i}-{\sum }_{i}^{N}{q}_{i}/N$. The reason why the mean charge deviates from zero is due to the tiny training sizes. Nevertheless, the mean-adjusted charges are very close to the ground truth of  +1 e/ −1 e molecular charges, despite the fact that the MLIP training is agnostic about these charge states. Even though the atomic charges are not quantitative due to the minimal training set, the learned charges are broadly consistent with chemical intuitions: The two under-coordinated oxygen atoms in ${{{\rm{C}}}}_{2}{{{\rm{O}}}}_{2}{{{\rm{H}}}}_{3}^{-}$ have the same strong negative charge, while the rest of the molecule is positively charged. The undercoordinated carbon in ${{{\rm{C}}}}_{3}{{{\rm{N}}}}_{3}{{{\rm{H}}}}_{10}^{+}$ has a positive charge, while the other atoms have smaller positive charges.

### Polar dipeptides

**Fig. 4:**
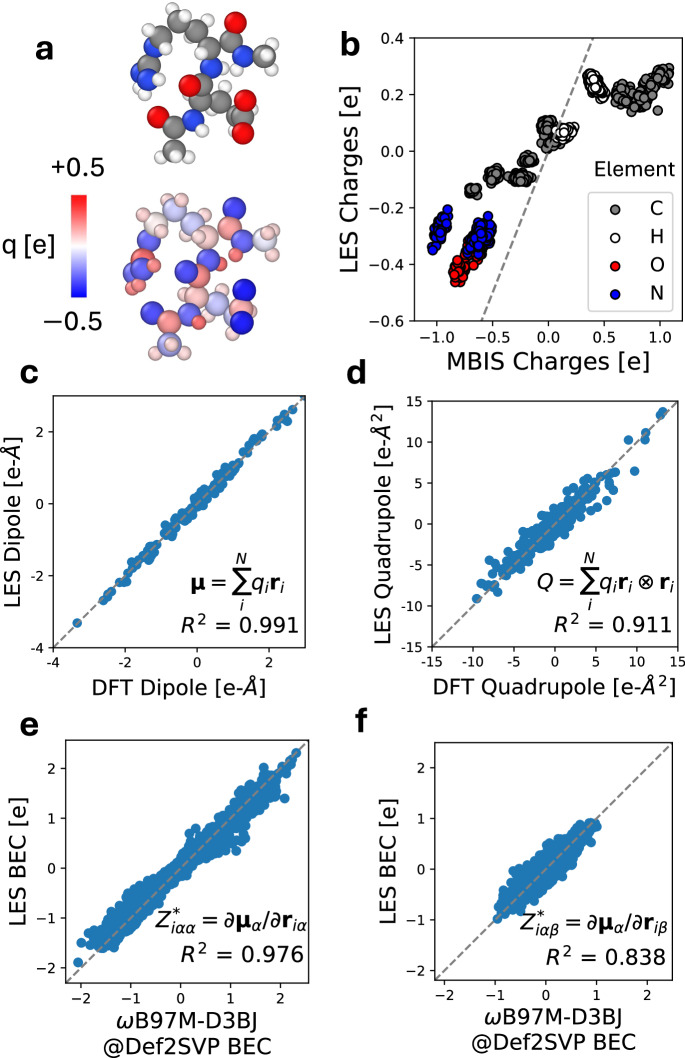
Benchmark of the latent Ewald summation (LES) model on the dipeptide test set. **a** Top: A snapshot of a dipeptide conformer from the test set. Bottom: The predicted charge (*q*) distribution. The color bar is in linear scale. **b** The predicted charges from LES compared to minimal basis iterative stockholder (MBIS) charges in SPICE^43^. **c** The predicted dipole components computed from the LES charges (${{\mathbf{\mu }}}=\mathop{\sum }_{i=1}^{N}{q}_{i}{{{\bf{r}}}}_{i}$) compared to the density functional theory (DFT) dipole components in SPICE. **d** The predicted traceless quadrupole components computed from the LES charges ($Q\,=\,\mathop{\sum }_{i=1}^{N}{q}_{i}{{{\bf{r}}}}_{i}\otimes {{{\bf{r}}}}_{i}$) compared to the DFT quadrupole components in SPICE. **e** The predicted diagonal born effective charge (BEC, ${Z}_{\alpha \alpha }^{*}=\partial {{{\boldsymbol{\mu }}}}_{\alpha }/\partial {{{\bf{r}}}}_{\alpha }$) components compared to BECs calculated with the *ω*B97M-D3BJ DFT functional in the Def2SVP basis. **f** The comparison for the off-diagonal BEC components (${Z}_{\alpha \beta }^{*}=\partial {{{\boldsymbol{\mu }}}}_{\alpha }/\partial {{{\bf{r}}}}_{\beta }$). Squared Pearson correlation coefficients *R*^2^ are shown in each plot where quantitative agreement is expected.

Since atomic charges in quantum mechanics are not well-defined quantities, a key question is whether the LES charges can be used to predict physical observables such as dipole and quadrupole moments. To answer this question, we turn to the SPICE dataset^43^, which contains DFT dipole and quadrupole moments as well as minimal basis iterative stockholder (MBIS) charges^44^ for a wide array of drug-like molecules. Specifically, we fit CACE-LR on a dataset of polar dipeptides, just by learning from the energy and forces. Then we determine whether LES is able to infer the DFT dipole and quadrupole moments on a holdout test set of unseen polar dipeptides (illustrated in Fig. 4a). We compute the predicted LES dipole via ${{\mathbf{\mu }}}=\mathop{\sum }_{i}^{N}{q}_{i}{{{\bf{r}}}}_{i}$ and quadrupole via $Q=\mathop{\sum }_{i}^{N}{q}_{i}{{{\bf{r}}}}_{i}\otimes {{{\bf{r}}}}_{i}$ where *q*_*i*_ are the charges predicted by LES and **r**_*i*_ are the positions of atoms *i*. To make the comparison translationally invariant, we additionally subtract the trace from the calculated and DFT quadrupole moments (${Q}^{{\prime} }=Q-\frac{1}{3}{{\rm{Tr}}}(Q)I$).

Figure 4b compares the charges predicted by LES to the MBIS charges from SPICE. As is seen, the charges predicted by LES correlate well with the MBIS charges, and agree with the usual ordering of electronegativities (O > N > C > H). However, we note that such agreement can only be qualitative (*R*^2^ = 0.87, MAE = 0.24). The reason behind this is that there is no rigorous definition of atomic charge^45^. To show this, we also compare between different definitions of DFT charges (MBIS, Mulliken charges, and Hirshfeld charges), as illustrated below in the Methods section. Indeed, the extent of disagreement between the LES charges and any definition of these DFT charges is similar to that between different definitions of DFT charges.

To evaluate the quality of the LES charges quantitatively, Fig. 4c compares the dipole moments (a well-defined experimental observable) derived from LES to that from DFT. Remarkably, we find that the derived dipoles from the LES charges are in excellent agreement with those from DFT (*R*^2^ = 0.991), even though the LR model is not trained explicitly on any charge or dipole information. In absolute terms, the LES mean absolute error (MAE) for dipole moments is 0.089 e-Å, comparable to the 0.063 e-Å MAE of MBIS charges derived directly from DFT densities. Figure 4d compares the calculated quadrupole moments to those of DFT. Again, we see good agreement of the LES quadrupoles with the physical DFT values (*R*^2^ = 0.911).

Furthermore, we compared the Born effective charge (BEC) tensor^36^, another well-defined physical quantity that corresponds to the derivative to the dipole moment with respect to atomic positions, i.e. ${Z}_{i\alpha \beta }^{*}=\frac{\partial {\mu }_{\alpha }}{\partial {r}_{i\beta }}$. Figure 4e and 4f compare the BECs predicted using the LES charges to the DFT reference values, for both the diagonal (*R*^2^ = 0.976) and off-diagonal (*R*^2^ = 0.838) BEC elements. There is again good agreement between the LES BECs and the DFT values. Overall, the agreement between DFT and LES dipoles, BECs, and quadrupoles shows that LES is able to convincingly model observables of the molecular charge density even though no charge information is explicitly input into the model training.

Again, we emphasize that DFT partial charges, such as MBIS, Mulliken charges, and Hirshfeld charges, are not physical observables – although there is significant disagreement between the such DFT charges and LES charges (Fig. 4b), there are similar disagreement between the different flavors of DFT charges. Nevertheless, they are all good predictors of the observable molecular dipole and quadrupole moments, as shown below in the Methods section. In other words, the LES charges are just as physical as any definitions of DFT partial charges. The ability of LES to infer dipole and quadrupole moments as well as BECs just from energies and forces strongly supports the thesis that it is not necessary to explicitly learn a specific definition of DFT charges or electronegativities.

### Dataset with different charge states and charge transfer

**Fig. 5:**
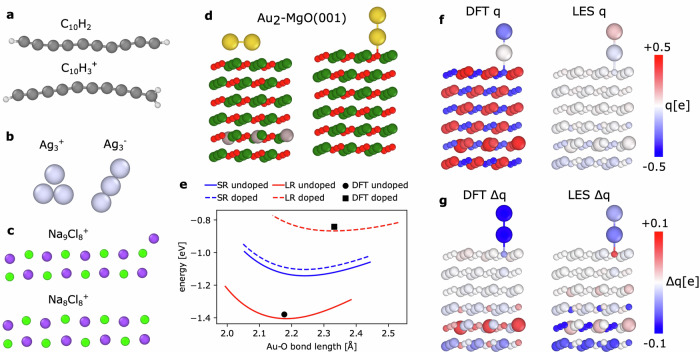
Illustrations and analyses of the four systems taken from ref. ^12^ with different charge states and charge transfer. Atom colors are as follows: H (white), C (gray), O (red), Na (purple), Mg (dark green), Cl (light green), Ag (silver), and Au (gold). **a** The ${{{\rm{C}}}}_{10}{{{\rm{H}}}}_{2}/{{{\rm{C}}}}_{10}{{{\rm{H}}}}_{3}^{+}$ set. **b** The ${{{\rm{Ag}}}}_{3}^{+/-}$ set has Ag trimers in positive or negative charge states. **c** The ${{{\rm{Na}}}}_{8/9}{{{\rm{Cl}}}}_{8}^{+}$ set. **d** The Au_2_-MgO(001) set has a wetting (left) or unwetting (right) Au_2_ on the doped (left) or undoped (right) MgO(001) surface. **e** Potential energies for the Au_2_ cluster adsorbed at the MgO(001) substrate for the non-wetting geometry for the Al-doped and undoped cases. SR and LR stand for short-range and long-range models. The equilibrium density functional theory (DFT) bond lengths, DFT energy and the associated minimum energies are denoted in black symbols. The Au-O bond length is the minimum distance between Au and O atoms. **f** The atomic charges (*q*) from the underlying DFT data (left), and the predicted atomic charge from Cartesian atomic cluster expansion long-range (CACE-LR, right) for the nonwetting Au_2_ cluster adsorbed on the doped MgO(001) substrate. **g** The change of atomic charges (*Δq*) due to doping, from the DFT data (left), and the predicted atomic charge from CACE-LR (right). The color bars are in linear scale.

Ko et al.^12^ compiled four datasets (${{{\rm{C}}}}_{10}{{{\rm{H}}}}_{2}/{{{\rm{C}}}}_{10}{{{\rm{H}}}}_{3}^{+}$, ${{{\rm{Ag}}}}_{3}^{+/-}$, ${{{\rm{Na}}}}_{8/9}{{{\rm{Cl}}}}_{8}^{+}$, and Au_2_ on MgO(001), illustrated in Fig. 5) that specifically target systems in different charge states or where charge transfer mediated by long-range electrostatic interactions is significant. In Table 1, we compare the CACE-LR errors with the values obtained with CACE-SR, 3G-HDNNP and 4G-HDNNP^12^, as well as a charge constraint ACE model through a local many-body expansion (*χ*+*η*(ACE))^46^. The comparison between CACE and ACE is a rather direct one: their descriptors are mathematically equivalent^47^. 4G-HDNNP and *χ*+*η*(ACE) both fit charges explicitly, while CACE-LR only fits to energy and forces and no total charge constraint was used. We used a 90% train and 10% test split, consistent with ref. ^12^.

**Table 1:** Test root mean squared errors (RMSE) energies (*E*) in meV/atom, forces (*F*) in meV/Å are reported for different models with cutoffs *r*_cut_

		ACE	*χ* + *η*(ACE)	3G-HDNNP	4G-HDNNP	CACE-SR	CACE-LR
	*r*_cut_	6 Å	6 Å	4.23 Å	4.23 Å	4.23 Å	4.23 Å
${{{\rm{C}}}}_{10}{{{\rm{H}}}}_{2}/{{{\rm{C}}}}_{10}{{{\rm{H}}}}_{3}^{+}$	E	0.76	0.75	2.045	1.194	1.27	0.73
	F	37.22	35.16	231.0	78.00	91.0	36.9
	*r*_cut_	6 Å	6 Å	5.29 Å	5.29 Å	5.29 Å	-
${{{\rm{Ag}}}}_{3}^{+/-}$	E	809.62	0.21	320.2	1.323	0.162	-
	F	285.81	23.10	1913	31.69	29.0	-
	*r*_cut_	6 Å	6 Å	5.29 Å	5.29 Å	5.29 Å	5.29 Å
${{{\rm{Na}}}}_{8/9}{{{\rm{Cl}}}}_{8}^{+}$	E	1.55	0.71	2.042	0.481	1.58	0.21
	F	41.72	12.35	76.67	32.78	48.8	9.78
	*r*_cut_	6 Å	6 Å	-	4.23 Å	5.5 Å	5.5 Å
Au_2_-MgO(001)	E	2.56	1.63	-	0.219	2.25	0.073
	F	88.70	50.27	-	66.00	59.3	7.91

The 3G-HDNNP and 4G-HDNNP values are from ref. ^12^. The values for the atomic cluster expansion (ACE) model and the charge constrained model (*χ* + *η*(ACE)) are from ref. ^46^. CACE-SR and CACE-LR stand for short-ranged and long-ranged Cartesian atomic cluster expansion (CACE) models. Short-ranged CACE with embedded charge states was used for the ${{{\rm{Ag}}}}_{3}^{+/-}$ system.

The ${{{\rm{C}}}}_{10}{{{\rm{H}}}}_{2}/{{{\rm{C}}}}_{10}{{{\rm{H}}}}_{3}^{+}$ set contains carbon chains terminated with hydrogen atoms in the neutral or positively charged state. With and without the added proton on the right-hand side of Fig. 5a, the atoms in the left half of the molecule can have almost identical environments but different atomic charges, which results in high fitting errors in 3G-HDNNP^12^ due to the contradictory information.

The ${{{\rm{Ag}}}}_{3}^{+/-}$ example illustrated in Fig. 5b contains Ag trimers in two different charge states. As the system size is small such that there are no long-range interactions, we used only a short-ranged CACE MLIP with embedded charge states. Since the energies depend on the overall charge states of the clusters, this causes the degeneracy issue between atomic structures and potential energy surfaces, leading to the poor performance of the 3G-HDNNP and the charge-agnostic ACE methods. Both the charge constraint *χ*+*η*(ACE) model and the charge-state-embedded CACE lift such degeneracies, leading to drastically improved descriptions.

The ${{{\rm{Na}}}}_{8/9}{{{\rm{Cl}}}}_{8}^{+}$ set (Fig. 5c) contains the ionic ${{{\rm{Na}}}}_{9}{{{\rm{Cl}}}}_{8}^{+}$ clusters and ${{{\rm{Na}}}}_{8}{{{\rm{Cl}}}}_{8}^{+}$ when a neutral Na atom is removed. This is also an example where global charge transfer is present. CACE-LR achieves the lowest errors in this case.

The Au_2_ − MgO(001) set (Fig. 5d) has a diatomic gold cluster supported on the MgO(001) surface with two adsorption geometries: an upright non-wetting orientation of the dimer attached to a surface oxygen, and a parallel wetting configuration on top of two Mg atoms. Moreover, three Al dopant atoms were introduced into the fifth layer below the surface (the gray atoms in the left panel of Fig. 5d). Despite having large distances of more than 10 Å, the dopant atoms have a major influence on the electronic structure and the relative stability between the wetting and the non-wetting configurations.

In this example, CACE-LR achieves errors that are approximately an order of magnitude smaller than those of the other methods compared. As an additional test, we performed geometry optimizations of the positions of the gold atoms, with the substrate fixed, for both doped and undoped surfaces. The results were compared to reference DFT calculations and previous results using the 4G-HDNNP method^12^. Note that the reference DFT results have been updated using tighter convergence settings of the geometry optimization, as performed by the authors of ref. ^12^. For the pure MgO substrate, the non-wetting configuration is energetically favored, whereas doping stabilizes the wetting geometry. The energy differences between the wetting and non-wetting configurations for both doped and undoped substrates are presented in Table 2. Short-range models, such as 2G-HDNNP and CACE-SR, predict nearly degenerate energy values for these configurations, as expected. In contrast, CACE-LR delivers highly accurate predictions, closely matching the reference results. Consistent with findings in ref. ^12^, we also present the potential energy surface for the non-wetting geometry on doped and undoped substrates as a function of the distance between the bottom Au atom and its neighboring oxygen atom, shown in Fig. 5e. Equilibrium bond lengths and energies derived from DFT are marked with black symbols. Notably, CACE-LR accurately resolves the distinct equilibrium bond lengths, with a slight shift in the potential energy surface likely attributable to differences in DFT convergence settings.

**Table 2:** Energy difference (*E*_wetting_ − *E*_nonwetting_) in meV between the wetting and nonwetting configurations for doped and undoped substrates

	DFT	2G-HDNNP	CACE-SR	4G-HDNNP	SevenNet 3-layers	SevenNet 4-layers	CACE-LR
Doped	-66.9	375	431	-41	141	8	-70.6
Undoped	934.8	375	431	975	721	898	931.3

The density functional theory (DFT), 2G-HDNNP and 4G-HDNNP values are from ref. ^12^. The SevenNet results are from ref. ^74^. CACE-SR and CACE-LR are our Cartesian atomic cluster expansion (CACE) short-range and long-range models.

We rationalize why the CACE-LR method delivers significantly more accurate predictions compared to other long-range methods that explicitly fit atomic charges. In Fig. 5f, we compare the atomic charge distribution from the underlying DFT data, obtained via Hirshfeld population analysis^12,48^, with the charges predicted by CACE-LR. The charges from CACE-LR are generally much smaller in magnitude and are primarily localized on the Au dimer and the dopant. In contrast, the DFT charges show sharp positive values for metal atoms and sharp negative values for oxygen atoms in the substrate. We hypothesize that explicitly modeling such DFT-derived charges for metals and oxygen is unnecessary for accurately predicting energy and forces. Short-ranged MLIPs are already well-suited to describe bulk oxides without dopants due to the screening effects that diminish the influence of these charge extremes. In Fig. 5g, we plot the changes in atomic charges resulting from doping, by taking the atomic charge difference for each atom from relaxed doped and doped structures, which shows a clear correlation between DFT and CACE-LR results. This example suggests that the charges predicted by CACE-LR can be interpreted as response charges rather than DFT partial charges, focusing on the aspects of charge redistribution relevant to energy and force predictions.

### Electrolyte/solid interfaces

As example applications to electrolyte/solid interfaces, we selected two sets of systems. The first is the Pt(111)/KF(aq) interface dataset from ref. ^49^, which describes the Pt electrode with the (111) surface forming an interface with K and F ions in water solutions. For training the MLIP, ref. ^49^ used a DPLR model: the short-ranged part is a standard Deep Potential (DP) model with a cutoff of 5.5 Å, and the long-range electrostatics is computed using spherical Gaussian charges associated with the nuclei (i.e., 6 e, 1 e, 9 e, 7 e, and 0 e for O, H, K, F, and Pt atoms, respectively) and the average positions of the MLWCs^10^ with a total charge of  −8 e associated with each O, K, and F atom. Note that such MLWC schemes are not applicable to conductors, so ref. ^49^ used the classical Siepmann-Sprik model^4^ to describe the Pt electrode in MD simulations.

The second dataset from ref. ^50^ is for modeling the anatase TiO_2_ (101) surface in contact with NaCl-water electrolyte solutions at various pHs. This dataset comprehensively spans the configurational space of bulk anatase TiO_2_, water, and various aqueous electrolyte solutions (NaCl, NaOH, HCl, and their mixtures), as well as anatase (101) interfaces with each of these liquids. ref. ^50^ trained a standard short-ranged DP and a DPLR MLIP. The LR part in the DPLR model is also based on the electrostatics of spherical Gaussian charges associated with the ions (nuclei + core electrons) and the valence electrons. More specifically, 4 e, 1 e, 6 e, 9 e, and 7 e for Ti, H, O, Na, and Cl ions, and each O, Na, and Cl ion has four WCs each carrying -2e.

We fitted the CACE-SR and CACE-LR models, without message passing. The results are presented in Table 3. We speculate that the improved performance of the CACE models compared to the DP models can be attributed to two reasons: First, the DP descriptors are restricted to two-body and three-body terms, while the ACE framework can include higher-body-order interactions and in this case we truncate to four-body terms. The inclusion of higher-body terms makes the model more expressive and helps alleviate the degeneracy problem^51^. Second, the LES scheme allows each atom to carry a flexible learned charge, in contrast with the fixed charge in the DPLR method.

**Table 3:** Test root mean squared errors (RMSE) are reported for energies (E) in meV/atom, forces (F)in meV/Å for different models with cutoffs *r*_cut_

		DPSR	DPLR	CACE-SR	CACE-LR
	*r*_cut_	-	5.5 Å	5.5 Å	5.5 Å
Pt(111)/KF(aq)	E	-	1.305	0.863	0.309
	F	-	75.00	58.6	34.1
	*r*_cut_	6 Å	6 Å	5.5 Å	5.5 Å
TiO_2_(101)/NaCl+NaOH+HCl(aq)	E	0.88	0.79	0.721	0.435
	F	124	119	103	70.5

The DPLR results for the Pt(111)/KF(aq) set are from ref. ^49^, and DPSR and DPLR results for the TiO_2_(101)/NaCl+NaOH+HCl(aq) set are from ref. ^50^. CACE-SR and CACE-LR are our Cartesian atomic cluster expansion (CACE) short-range and long-range models.

**Fig. 6:**
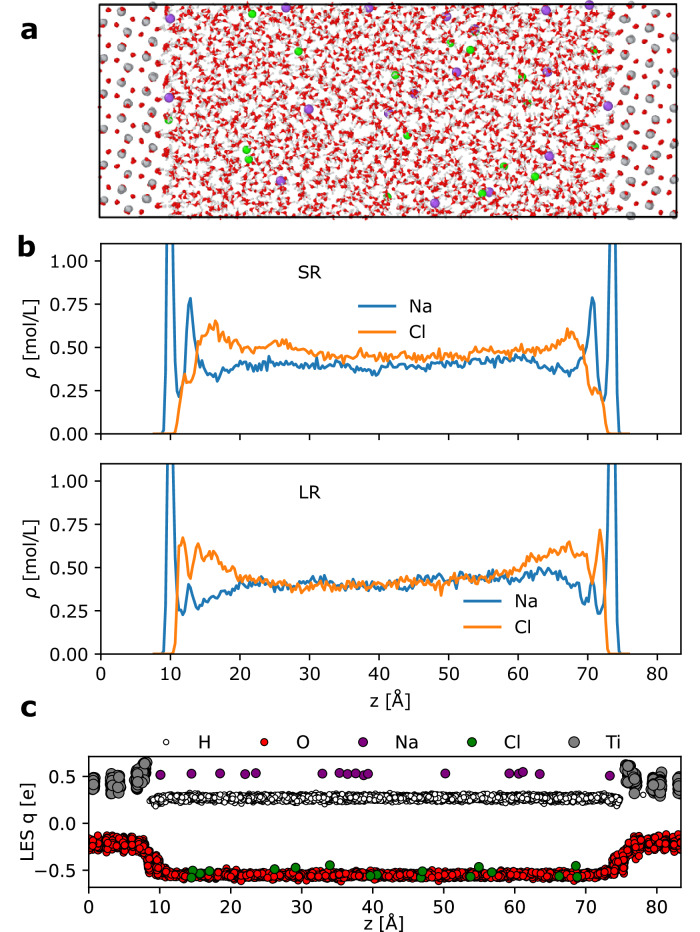
Illustration and analysis of the electrolyte/solid interface system of the anatase TiO_2_ (101) surface and NaCl in water solution. **a** A representative snapshot of the anatase TiO_2_ (101) surface and NaCl in water solution, randomly selected from the training set. Atom colors are as follows: H (white), O (red), Na (purple), Cl (green), and Ti (gray). **b**, **c** Plane-averaged ion density *ρ* (**b**) and predicted latent Ewald summation (LES) charges *q* on atoms (**c**) along the *z*-direction (normal to the surface) for the TiO_2_-NaCl solution interface obtained from short-range (SR) and long-range (LR) machine learning interatomic potential MD simulations at 600 K.

To showcase the effect of long-range interactions on the structures of the electrolyte and the electric double layer (EDL), we performed MD simulations at 600 K for 5 ns on a large system of anatase TiO_2_ surface and NaCl in water solution (illustrated in Fig. 6). This is also a test that was performed in ref. ^50^. Figure 6b shows the ion distributions obtained from the MD simulations using the CACE-SR and CACE-LR models. In reality, the solution should recover its bulk properties in the central region that is away from the interface and have equal densities of Na^+^ and Cl^−^ ions. However, the SR model, lacking long-range electrostatic interactions, imposes no energy penalty for unphysical charge imbalances. Consequently, the MD simulation predicts an excess Cl^−^ density of approximately 0.05 mol/L in the center of the box. In contrast, incorporating long-range interactions with the CACE-LR model eliminates this artifact and alters the ion distributions within the EDL. These effects, including the correction of charge imbalance and modified EDL structures, were also reported in ref. ^50^. Notably, the CACE-LR model predicts a significantly lower second Na^+^ density peak near the interface compared to ref. ^50^. Figure 6c shows the predicted LES charges on atoms at different positions. Mostly notably for oxygen (red symbols) and titanium (gray symbols), the magnitude of these charges are dependent on whether the atoms are in the bulk region or at the interface. Such variance can be understood as coming from the difference of polarization environments. and may help capture the complex electrostatic interactions in interfacial systems.

### Solid-solid interface

Atomistic modeling of solid-solid interfaces is essential in understanding material synthesizability^52^. The heterogeneous nature of these interfaces requires long-period structures, particularly in cases involving charge transfer, which necessitates long-range descriptions beyond standard MLIPs. To evaluate the predictive accuracy of our models, we conducted a benchmark study comparing CACE-SR and CACE-LR using the LiCl(001)/GaF_3_(001) interfacial system^53^. The training dataset includes bulk and interfacial configurations in the LiCl-GaF_3_ chemical space with corresponding DFT-calculated energies and interatomic forces. To assess model uncertainty, we trained an ensemble of four SR/LR models and used their predictions to estimate force uncertainties (see Methods). For in-distribution (ID) test set performance, CACE-SR and CACE-LR models achieve RMSEs of 78.8 meV/Å and 67.8 meV/Å, respectively.

**Fig. 7:**
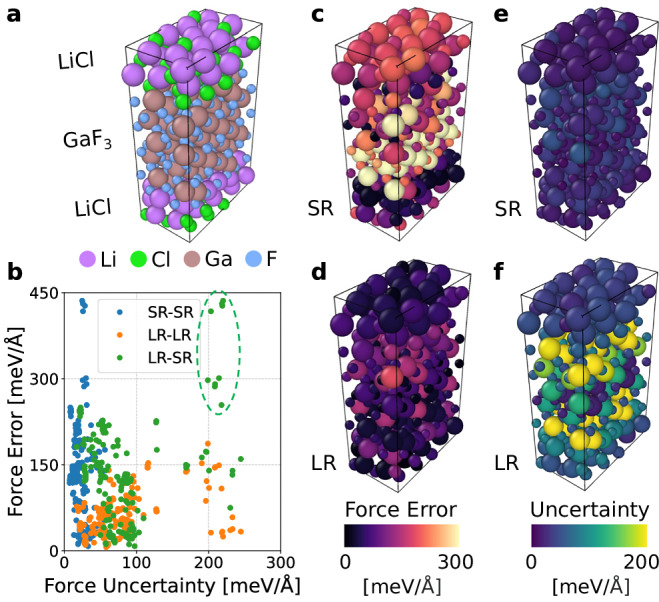
Illustration and analysis of the system of the LiCl(001)/GaF_3_(001) interface. **a** A density functional theory (DFT)-relaxed structure of the LiCl(001)/GaF_3_(001) interface. **b** Correlation between force errors and uncertainties computed from ensemble predictions. SR and LR stand for short-range and long-range models. Blue: SR model error vs. SR model uncertainty; Orange: LR model error vs. LR model uncertainty; Green: SR model error vs. LR model uncertainty. The circled region corresponds to the high-error region in (**c**) from Cartesian atomic cluster expansion (CACE) short-range (CACE-SR) and high-uncertainty region in (**f**) from CACE long-range (CACE-LR). **c**−**f** Atomic-resolved force errors (left panels) and uncertainty estimates (right panels) for SR (top) and LR (bottom) models.

To evaluate model transferability, we constructed an out-of-distribution (OOD) test set using a large solid-solid heterostructure relaxed with DFT calculations (~30 Å in the *z*-direction, Fig. 7a). This extended structure, containing eight Ga layers and four Li layers, represents a more realistic interface with much reduced finite-size effects compared to the training configurations. On this OOD set, the LR model demonstrates improved predictive accuracy with a force component error of 40.5 meV/Å compared to 116.3 meV/Å for the SR model. The atomic-resolved force errors are visualized in Fig. 7c, d, which were computed from the square root of the sum of force component errors in *x*, *y*, *z*-directions.

Force uncertainties were quantified using ensemble variance from the four trained models. The SR model exhibits lower uncertainties (Fig. 7e), indicating a good parametrization on the ID training set. In contrast, the LR model shows elevated uncertainties (Fig. 7f), effectively identifying OOD atomic environments in the heterostructure. The correlation between the absolute force errors (RMSE against DFT) and uncertainties is shown in Fig. 7b, where green dots specifically highlight the relationship between SR model errors (poor prediction) and LR model uncertainties (OOD detection). Interestingly, the LR model identifies regions of SR model failure (green dashed circle in Fig. 7b), which are further evidenced by the spatial correspondence in Fig. 7c, f. These results suggest that despite the SR MLIPs achieving adequate ID performance for this system, they lack the mathematical framework to capture long-period structure features that are essential for electrostatic interactions. In contrast, the LR models with LES overcome this limitation with improved transferability. More generally, the enhanced OOD detection capabilities are essential for robust uncertainty quantification in broader applications such as materials property predictions and generation^54^. While our current implementation relies on computationally intensive ensemble variance, the LES framework is compatible with various uncertainty quantification methods, including Gaussian mixture models^55^, Monte Carlo dropout^56^, and deep evidential regression^57^.

## Discussion

The LES framework is highly interpretable in physical terms: the hidden variable *q*, when restricted to one dimension for computing electrostatic long-range potentials, corresponds to the partial charges for describing electrostatic interactions. In cases such as random charges and electrolyte solutions, where the underlying potential energy surfaces are described by classical forcefields with fixed charges, LES accurately recovers those charges. For quantum-mechanical systems, such as those described using DFT, the LES-derived partial charges can be understood as a coarse-grained approximation of the net electrostatic effect of electron density polarization. This approximation has also been rationalized and applied to parameterize scaled charges in classical forcefields^3^.

Notably, atomic charges in quantum-mechanical systems are not physical observables. In DFT, there exists a wide variety of methods to assign local atomic charges given the global charge density, each providing different frameworks and values^45,58^. These include Mulliken population analysis that relies on the overlap of atomic orbitals^59^, shareholder methods such as Hirshfeld population analysis^48^ and MBIS^44^, fitted atomic charges to the electrostatic potential with restraints^60^, and parameterizations based on these schemes^45^. For polar dipeptides, as shown in Fig. 4, LES charges are correlated with, but not equivalent to, several definitions of DFT charges such as MBIS charges, Mulliken charges, and Hirshfeld charges. Meanwhile, similar levels of discrepancy exist between the different flavors of the DFT charges. Yet, despite this imperfect correlation, LES charges reproduce DFT dipoles and quadrupoles with remarkable accuracy. Moreover, the LES charges are able to reproduce the Born effective charges, which are physical quantities that measure how atoms in the system respond to an external electric field^36^. This free lunch–predicting dipole, quadrupole moments, and BECs without explicitly learning the multipoles or charges – highlights the physical interpretability embedded in the LES framework.

Indeed, the ambiguity of DFT partial charges suggests that directly learning such charges may not be necessary for – or may even be a detriment to – constructing accurate interatomic potentials. This insight is supported by results for four challenging systems involving different charge states and charge transfer (${{{\rm{C}}}}_{10}{{{\rm{H}}}}_{2}/{{{\rm{C}}}}_{10}{{{\rm{H}}}}_{3}^{+}$, ${{{\rm{Ag}}}}_{3}^{+/-}$, ${{{\rm{Na}}}}_{8/9}{{{\rm{Cl}}}}_{8}^{+}$, and Au_2_ on MgO(001)), where CACE-LR outperformed both 4G-HDNNP^12^ and *χ* + *η*(ACE)^46^, which explicitly learn charges and perform charge equilibration (see Table 1). For interfacial systems, such as Pt(111)/KF(aq) and TiO_2_(101)/NaCl + NaOH + HCl(aq), CACE-LR also achieved greater accuracy compared to DPLR, which learns the positions of Wannier centers (see Table 3).

One can further speculate that the improved performance of LES compared to the other methods stems from the fact that LES does not directly learn from charges. For instance, in the Au_2_-MgO system, LES achieves an error an order of magnitude lower than 4G-HDNNP^12^ and *χ* + *η*(ACE)^46^. This likely results from LES capturing the response charge-changes in atomic charges due to doping-rather than the sharply peaked and method-dependent DFT charges, as illustrated in Fig. 5f,g. While our test uses simple metal oxides, the response charge formalism is particularly relevant for complex ionic systems, such as transition metal oxides. Previous studies have shown that materials with localized *d*-electrons exhibit self-regulating response in DFT^61^, where the system maintains constant local charges on transition metal atoms by minimizing external perturbations through rehybridization^62^. Given this complexity and the fact that DFT charges vary depending on the computation method, directly inferring them introduces inefficiencies in resolving their ambiguous components^63^. The strong performance of LES suggests that the detailed prediction of atomic charges is less critical; instead, the primary focus should remain on accurately predicting physically observable quantities, such as energies and forces. Moreover, by avoiding the direct learning of charges, LES circumvents the need for explicit charge equilibration, thereby reducing the associated computational overhead.

Omitting long-range interactions can result in severely inaccurate predictions for many systems. For example, standard short-ranged MLIPs fail to predict the binding curve of a charged molecular dimer (Fig. 3), cannot distinguish the different adsorption behaviors of Au dimers on doped and undoped MgO substrates (Table 2 and Fig. 5e), and even produce a charge imbalance in the bulk region of the TiO_2_-NaCl(aq) solution interface (Fig. 6). Alarmingly, the commonly used ensemble uncertainty quantification method was unable to detect the large errors of SR MLIPs in out-of-distribution cases, such as the solid-solid LiCl(001)/GaF_3_(001) interface. This highlights that standard SR models can yield unphysical results in certain systems, and these errors may go unnoticed when relying solely on conventional uncertainty quantification techniques.

In summary, we thoroughly benchmarked the LES method, a physics-informed approach that learns long-range interactions directly from energies and forces, without requiring explicit charge labels or additional input. We show that LES can achieve better accuracy in energy and force predictions compared to methods that explicitly learn from DFT partial charges. Moreover, LES is able to learn the true underlying electrostatics: for classical systems with fixed charges, LES can reproduce these exact charges; for quantum mechanical systems, LES can infer dipole, quadrupole moments, and BECs. The framework consistently provides superior accuracy in modeling long-range interactions compared to existing MLIPs. We thus demonstrate LES to be a versatile and efficient tool for addressing a wide range of challenging systems where long-range interactions play a critical role, such as electrolyte interfaces, charged molecular complexes, and ionic solutions. In the future, we will incorporate LES into general-purpose MLIPs that are applicable for many systems across the periodic table.

## Methods

### Details on the MLIP training

#### Random charges

The dataset contains a total of 1000 configurations, and each configuration has 64 atoms with +1e charge and 64 atoms with -e charge. The set was collected from NPT simulations at 4000 K and zero external pressure. We performed the NPT simulations and computed the energy and forces in LAMMPS, using the Nose-Hoover thermostat and barostat. The standard deviations in energy and forces are 0.17 eV/atom and 2.0 eV/Å, respectively.

For the CACE representation, we used 6 Bessel radial functions with *c* = 12, ${\ell }_{\max }=3$, ${\nu }_{\max }=3$, *N*_embedding_ = 3, no message passing, and different cutoff of *r*_cut_ = 4.5 Å, 5 Å, or 5.5 Å. For the long-range component, we used a 1-dimensional *q*, *σ* = 1 Å, and a maximum cutoff of *k*_*c*_ = 2*π* (dl = 1 Å in the CACE LES syntax) in the Ewald summation.

#### Electrolyte solution

The dataset of KF aqueous solution contains both bulk electrolyte solution configurations (1206 configurations with 64 water molecules and 0–5 ion pairs), and electrolyte-vapor interfaces (603 configurations with 225 water molecules and 1, 2, or 10 ion pairs). We performed NVT MD simulations at 370 K to collect snapshots using the Nose-Hoover thermostat in LAMMPS, employing SPC/Fw water^38^ (O has charge −0.8476 e, H has charge +0.4238 e), and ions with fixed charges (K has charge +1e, F has charge -1e) and Lennard-Jones interactions^39^. The standard deviations in energy and forces are 0.074 eV/atom and 0.9 eV/Å, respectively.

For the CACE representation, we used 6 Bessel radial functions with *c* = 12, ${\ell }_{\max }=3$, ${\nu }_{\max }=3$, *N*_embedding_ = 4, no message passing (*T* = 0) or one message passing layer (*T* = 1), and different cutoffs of *r*_cut_ = 4.5 Å, or 5.5 Å. For the long-range component, we used a 1-dimensional *q*, *σ* = 1 Å, and a maximum cutoff of *k*_*c*_ = *π* (dl = 2 Å) in the Ewald summation.

**Fig. 8:**
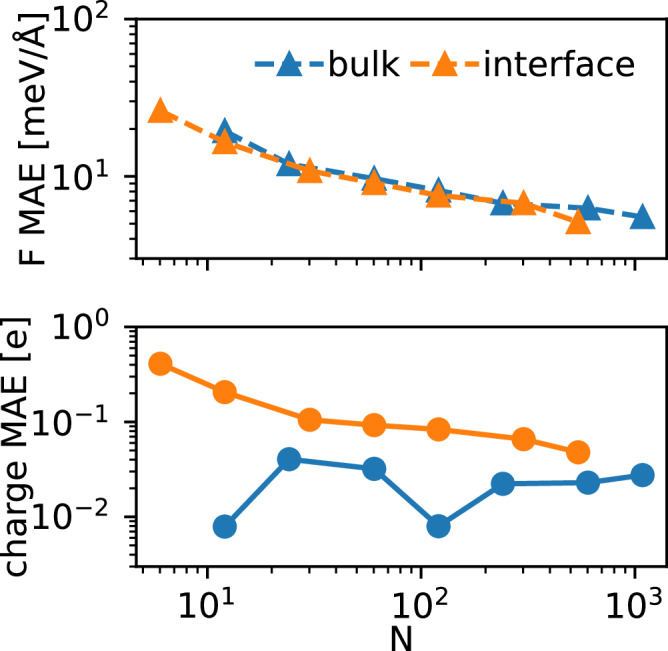
Learning the efficiency of Cartesian atomic cluster expansion (CACE) long-range (CACE-LR) models trained only on bulk electrolyte solution or electrolyte-vapor interfacial configurations of potassium fluoride (KF) aqueous solution. The mean absolute errors (MAEs) on forces (F) and charges (*q*) are shown for CACE-LR models. Both panels display the model performance as a function of training set size (*N*) for each configuration type.

In Fig. 8 we show the learning curves from learning using only bulk electrolyte solution configurations, or only electrolyte-vapor interfacial configurations. Interestingly, the learning efficiency for forces is almost identical for the two sets, but the charges are more difficult to learn from the interfacial systems.

#### Charged molecular dimers

The LODE molecular dimer dataset includes energy and force information calculated using the HSE06 hybrid density functional theory (DFT) with a many-body dispersion correction. We used the molecular pair with id 0.

The CACE representation uses a cutoff *r*_cut_ = 5 Å, 6 Bessel radial functions, *c* = 8, ${\ell }_{\max }=2$, ${\nu }_{\max }=2$, *N*_embedding_ = 3, and one message passing layer (*T* = 1). The long-range component *E*^lr^ employed a 1-dimensional hidden variable computed from the same CACE *B*-features and utilized Ewald summation with *σ* = 1 Å and a **k**-point cutoff of *k*_*c*_ = 2*π*/3 (dl = 3 Å).

#### Polar dipeptides

The dataset of polar dipeptides was taken from the SPICE dataset developed by Eastman et al.^43^. The dataset contains energies and forces for a large number of drug-like molecules, including a complete set of dipeptides formed from 26 amino acid variations. The subset used in Fig. 4 consists of dipeptides with one positively charged amino acid (arg, lys, or hip) and one negatively charged amino acid (glu or asp), resulting in a total of 12 dipeptides (with both ways of bonding together two amino acids included, e.g., glu-arg or arg-glu) with 50 conformers each. We retain the conformers of one of the 12 dipeptides as a test set and 10% of the remaining structures as a validation set. Born effective charges are not available in SPICE and so were calculated with the same functional (*ω*B97M-D3BJ^64,65^) in PySCF^66^ version 2.8.0 with version 0.1.0 of the properties module using the smaller Def2SVP basis^67^.

Table 4 shows the RMSE performance of both CACE-LR and CACE-SR in determining the energies and forces of these dipeptides. CACE-LR provides slightly better forces and errors than CACE-SR as well as better generalizability to the conformers of the unseen dipeptide (glu-arg). For the CACE model, we used *r*_*cut*_ = 4.0 Å, 6 trainable Bessel radial functions, *c* = 12, ${\ell }_{\max }=4$, ${\nu }_{\max }=3$, one message passing layer (*T* = 1), and different embeddings of sender and receiver nodes with *N*_embedding_ = 4. For LES, we used *σ* = 1.5 Å and the long-range energy from Eq. (2) was computed in real space as the configurations are with aperiodic conditions.

**Table 4:** Performance of Cartesian atomic cluster expansion (CACE) short-range (CACE-SR) and CACE long-range (CACE-LR) on the validation and test sets of the 12 polar dipeptides

	CACE-SR	CACE-LR	CACE-SR	CACE-LR
	Val	Val	Test	Test
E	1.97	1.29	2.35	1.88
F	58.82	53.15	72.43	61.13

Errors are reported via root mean square error (RMSE) in meV/atom for energy (E) and in meV/Å for forces (F).

**Fig. 9:**
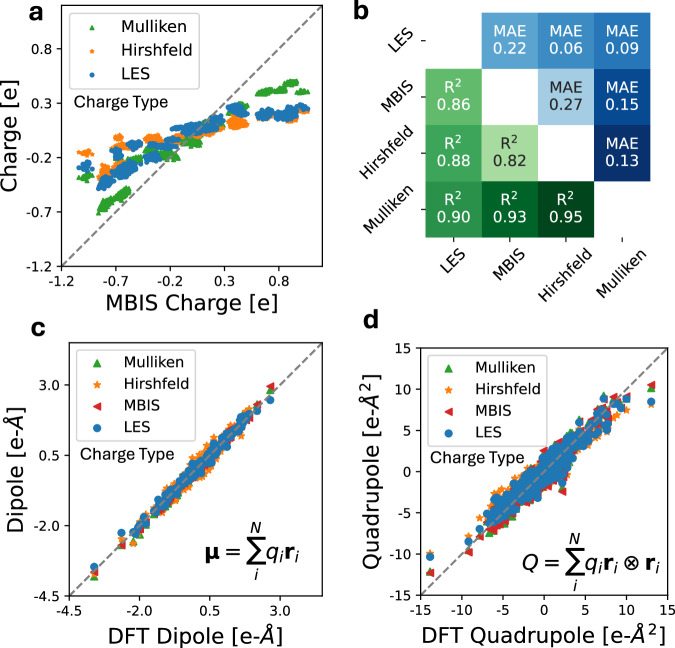
Comparison of latent Ewald summation (LES) and density functional theory (DFT) charges, molecular dipole and quadrupole moments. **a** Comparison of minimal basis iterative shareholder (MBIS) charges to Mulliken, Hirshfeld, and LES charges on the dipeptide validation set. **b** Matrix of Pearson *R*^2^ correlation (lower triangular) and mean absolute error (MAE) (upper triangular) between different partial charges. **c** The predicted dipole components computed from different partial charges (${{\mathbf{\mu }}}=\mathop{\sum }_{i=1}^{N}{q}_{i}{{{\bf{r}}}}_{i}$) compared to the DFT dipole components in SPICE^43^. **d** The predicted traceless quadrupole components computed from the MBIS charges ($Q=\mathop{\sum }_{i=1}^{N}{q}_{i}{{{\bf{r}}}}_{i}\otimes {{{\bf{r}}}}_{i}$) compared to the DFT quadrupole components in SPICE.

Figure 9 a and b compare LES charges to different partial charges derived from DFT on the validation set, including MBIS charges, Mulliken charges, and Hirshfeld charges. MBIS charges are taken from SPICE while Mulliken charges derived from meta-Löwdin atomic orbitals and Hirshfeld charges were calculated in PySCF in a smaller Def2SVP basis. As is seen, although there is a good correlation between all partial charges, the agreement is purely qualitative. Nevertheless, all partial charges show good agreement with the DFT dipoles and quadrupoles (Fig. 9c, d).

**Fig. 10:**
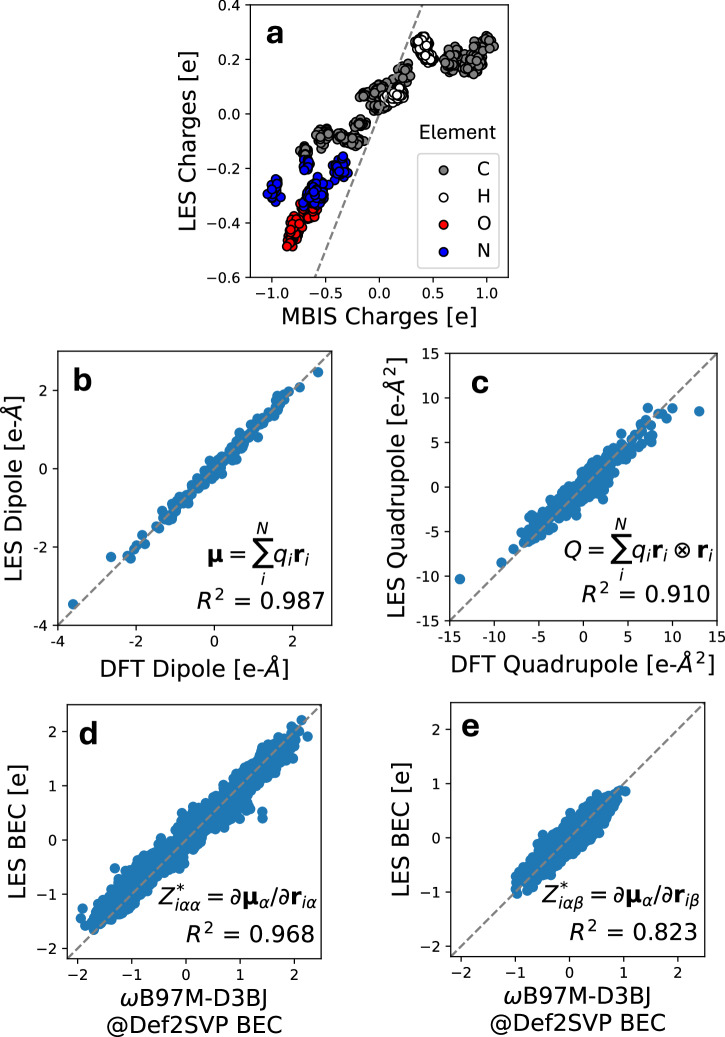
Results on the dipeptide validation set. **a** The predicted charges from latent Ewald summation (LES) compared to minimal basis iterative stockholder (MBIS) charges in SPICE^43^ on the validation set. **b** The predicted dipole components computed from the LES charges (${{\mathbf{\mu }}}=\mathop{\sum }_{i=1}^{N}{q}_{i}{{{\bf{r}}}}_{i}$) compared to the density functional theory (DFT) dipole components in SPICE on the validation set. **c** The predicted traceless quadrupole components computed from the LES charges ($Q=\mathop{\sum }_{i=1}^{N}{q}_{i}{{{\bf{r}}}}_{i}\otimes {{{\bf{r}}}}_{i}$) compared to the DFT quadrupole components in SPICE on the validation set. **d** The predicted diagonal born effective charge (BEC, ${Z}_{\alpha \alpha }^{*}=\partial {{{\boldsymbol{\mu }}}}_{\alpha }/\partial {{{\bf{r}}}}_{\alpha }$) components compared to BECs calculated with the *ω*B97M-D3BJ DFT functional in the Def2SVP basis on the validation set. **e** The comparison for the off-diagonal BEC components (${Z}_{\alpha \beta }^{*}=\partial {{{\boldsymbol{\mu }}}}_{\alpha }/\partial {{{\bf{r}}}}_{\beta }$) on the validation set.

Additionally, Fig. 10 shows the performance of CACE-LR in predicting dipoles and quadrupoles on the 55-configuration validation set. As is seen performance on the validation set is similar to that on the holdout test set (Fig. 4).

#### 4G-HDNNP dataset

The four datasets (${{{\rm{C}}}}_{10}{{{\rm{H}}}}_{2}/{{{\rm{C}}}}_{10}{{{\rm{H}}}}_{3}^{+}$, ${{{\rm{Ag}}}}_{3}^{+/-}$, ${{{\rm{Na}}}}_{8/9}{{{\rm{Cl}}}}_{8}^{+}$, and Au_2_ − MgO(001)) are from ref. ^12^.

For ${{{\rm{C}}}}_{10}{{{\rm{H}}}}_{2}/{{{\rm{C}}}}_{10}{{{\rm{H}}}}_{3}^{+}$, we used *r*_cut_ = 4.23 Å (8 Bohr) which is the same as the cutoff in ref. ^12^, 6 Bessel radial functions, *c* = 8, ${\ell }_{\max }=3$, ${\nu }_{\max }=3$, *N*_embedding_ = 2, no message passing, 1-dimensional hidden variable, *σ* = 1 Å, and *k*_*c*_ = *π* (dl = 2 Å).

For ${{{\rm{Ag}}}}_{3}^{+/-}$, we used *r*_cut_ = 5.29 Å (10 Bohr), 6 Bessel radial functions, *c* = 8, ${\ell }_{\max }=3$, ${\nu }_{\max }=3$, *N*_embedding_ = 1, no message passing, total charge state embedding, and no long-range component.

For ${{{\rm{Na}}}}_{8/9}{{{\rm{Cl}}}}_{8}^{+}$, we used *r*_cut_ = 5.29 Å (10 Bohr), 6 Bessel radial functions, *c* = 8, ${\ell }_{\max }=3$, ${\nu }_{\max }=3$, *N*_embedding_ = 2, no message passing, 1-dimensional hidden variable, *σ* = 1.5 Å, and *k*_*c*_ = 2*π*/3 (dl = 3 Å).

For Au_2_-MgO(001), we used *r*_cut_ = 5.5 Å, 6 Bessel radial functions, *c* = 12, ${\ell }_{\max }=3$, ${\nu }_{\max }=3$, *N*_embedding_ = 4, no message passing, 1-dimensional hidden variable, *σ* = 1 Å, and *k*_*c*_ = *π* (dl = 2 Å).

#### Electrolyte/solid interfaces

The Pt(111)/KF(aq) interface dataset from ref. ^49^ was computed at the PBE-D3 level of theory, and it contains 4687 configurations covering bulk KF/water electrolytes, KF/water electrolyte-vapor interfaces, and KF/water electrolyte-Pt(111) interfaces.

We used a random train/valid/test split of 3318/369/1000 configurations for training the CACE-SR and CACE-LR models. The CACE-SR model uses *r*_cut_ = 5.5 Å, 6 Bessel radial functions, *c* = 12, ${\ell }_{\max }=3$, ${\nu }_{\max }=3$, *N*_embedding_ = 5, and no message passing. The LR model uses a one-dimensional hidden variable, *σ* = 1 Å, and *k*_*c*_ = *π* (dl = 2 Å).

The TiO_2_(101)/NaCl+NaOH+HCl(aq) dataset from ref. ^50^ contains a total of 30103 configurations and spans a comprehensive range of gas phase water, bulk solutions, and TiO_2_, and interfacial configurations. The dataset was computed at the SCAN DFT level of theory and was collected through an active learning approach.

We used a random train/valid/test split of 24393/2710/3000 configurations for training the CACE-SR and CACE-LR models. The CACE-SR model uses *r*_cut_ = 5.5 Å, 6 Bessel radial functions, *c* = 12, ${\ell }_{\max }=3$, ${\nu }_{\max }=3$, *N*_embedding_ = 5, and no message passing. The LR model uses a one-dimensional hidden variable, *σ* = 1 Å, and *k*_*c*_ = *π* (dl = 2 Å).

To perform the MD simulation of the TiO_2_(101)/NaCl(aq) system, we used the same system setup as ref. ^50^: The periodic system, illustrated in Fig. 6, consisting of a five-layer (3 × 9) anatase (101) slab (540 TiO_2_ units) in contact with a 67 Å thick layer of aqueous electrolyte (2376 water molecules and 18 NaCl ion pairs). We used NVT ensemble at 600 K with the Nose-Hoover thermostat. The timestep was set to 1 fs, and we modified the hydrogen mass to 10. The total length was 5 ns.

#### Interphase of LiCl-GaF_3_

To generate the training dataset, we used Bayesian force fields implemented in the Flare package^68^ to sample the atomic configurations with on-the-fly (OTF) MD simulations of the interface structures of LiCl(001)/GaF_3_(001), which were generated with the CoherentInterfaceBuilder in pymatgen package^69^. The DFT calculation was called when the uncertainty threshold is higher than std_tolerance_factor=-0.04 in Flare. OTF-MD in the NVT ensemble was initiated from each strained configuration by heating from 0 K to the target temperatures (*T* = 600/1200 K). The DFT calculations were performed with VASP in the generalized gradient approximation (GGA) with PBE functional^70^, using a *k*-point mesh of 1000 per reciprocal atom and a plane-wave energy cutoff of 520 eV. The calculations were converged to 10^−6^ eV in total energy and the DFT-D3 method of Grimme was used to include Van der Waals corrections^65^. In total, 3339 DFT-calculated atomic configurations were collected and split into training/validation/test sets with a ratio of 8:1:1.

For the CACE representation, we used 6 Bessel radial functions with *c* = 8, ${\ell }_{\max }=3$, ${\nu }_{\max }=3$, *N*_embedding_ = 3, one message passing, and a cutoff of *r*_cut_ = 5.5 Å. For the long-range component, we used a one-dimensional *q*, *σ* = 1 Å, and a maximum cutoff of *k*_*c*_ = *π* (dl = 2 Å) in the Ewald summation.

The atomic-resolved force uncertainty was calculated as the root sum of variances along the Cartesian coordinates: $\sigma (F)=\sqrt{{\sigma }^{2}({F}_{x})+{\sigma }^{2}({F}_{y})+{\sigma }^{2}({F}_{z})}$. For each directional component, the variance *σ*^2^(*F*_*i*_) was computed across the ensemble of *N* = 4 models using ${\sigma }^{2}({F}_{i})=\frac{1}{N}\mathop{\sum }_{j=1}^{N}{({F}_{i}^{j}-{\bar{F}}_{i})}^{2}$, where ${F}_{i}^{j}$ represents the force prediction from the *j*-th model in direction *i* ∈ {*x*, *y*, *z*}, and ${\bar{F}}_{i}$ denotes the ensemble-averaged force in that direction.

### Implementation

We implemented the LES method using PyTorch, and the code is available in https://github.com/BingqingCheng/cace. The raw predicted hidden variables should be scaled by a factor of 1/9.48933 to obtain the LES charges for e.g. dipole moment prediction, due to the internal normalization factor used (1/2*ϵ*_0_ = 1).

In the current work, we have optimized the LES part of the code in the CACE repository: we now first add up the short-range and the long-range energies using a FeatureAdd module and then apply the autograd of the total energy with respect to atomic positions to obtain forces. For comparison, the previous implementation uses two autograd operations to obtain short-range and long-range forces separately and then sums up the forces^24^. The elimination of one autograd operation significantly reduces computational cost. Additionally, we made the current CACE model fully compatible with TorchScript, facilitating future deployment and integration in various platforms.

**Fig. 11:**
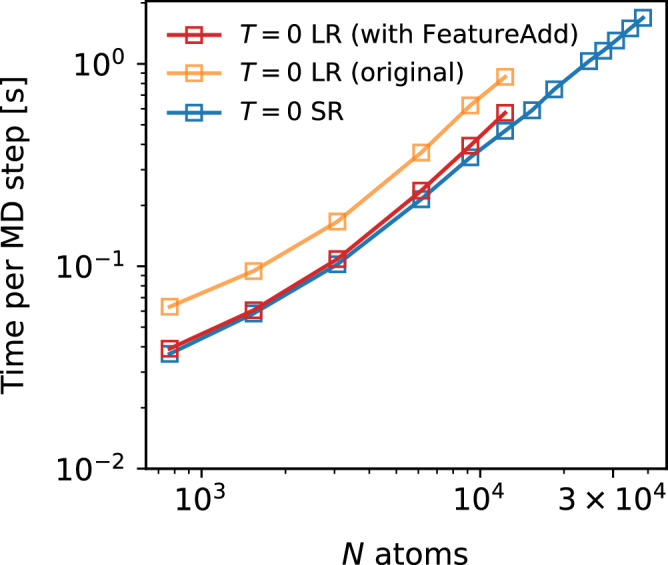
Computational performance benchmarks of molecular dynamics (MD) simulations. Timing of MD simulations of bulk liquid water for different system sizes (*N*) using different Cartesian atomic cluster expansion (CACE) models with no message passing (*T* = 0) was performed on an NVIDIA L40S GPU. SR and LR denote short-range and long-range models. Both axes are shown on a logarithmic scale.

To test the inference speed of the updated implementation, we benchmarked on water MLIPs with the same model parameters as our previous benchmark^24^. Namely, the CACE model uses *r*_cut_ = 5.5 Å, 6 Bessel radial functions, *c* = 12, ${\ell }_{\max }=3$, ${\nu }_{\max }=3$, *N*_embedding_ = 3, and no message passing (*T* = 0). The LR part uses a one-dimensional hidden variable, *σ* = 1 Å, and *k*_*c*_ = *π* (dl = 2 Å). The MLIPs are trained on the liquid water dataset from ref. ^71^. Figure 11 compares the speed of the two MLIP models (SR and LR) for MD simulations of liquid water on a single NVIDIA L40S GPU with 48 GB of memory. Figure 11 shows that the computational overhead of including long-range interactions is minimal using the updated implementation (red curve), and the performance becomes comparable to that of the SR model (blue curve). Moreover, all models show favorable scaling. The SR model here supports simulations with up to approximately 40,000 atoms on a single GPU, while the LR model supports up to around 13,000 atoms.

#### Notes on charge equilibration

Although in all the examples we tested, charge equilibration was not needed, we want to note that it is possible to fix the total charge while avoiding the charge equilibration. One possibility is to add the following penalty term to the total potential energy *E*:

5 $${E}^{\lambda }=\lambda {\left(Q-\mathop{\sum }_{i=1}^{N}{q}_{i}\right)}^{2},$$

where the positive constant *λ* can be understood as a Lagrangian multiplier, and *Q* is the referenced total charge of the system. Although we do not use this scheme in any of the examples, we provide it here for future use cases.

### Reporting summary

Further information on research design is available in the Nature Portfolio Reporting Summary linked to this article.

## Supplementary information

Supplementary Information

Reporting Summary

Transparent Peer Review file

## Source data

Source Data

## Data Availability

The training sets, training scripts, MD input files, and trained CACE potentials are available at https://github.com/BingqingCheng/cace-lr-fit; see ref. ^72^. Source data for all figures are provided with this paper. Source data are provided with this paper.

## Code availability

The CACE package is publicly available at https://github.com/BingqingCheng/cace; see ref. ^73^. The code is released under the MIT license. The long-range method is implemented as an Ewald module.
