# Supplementary material for: Machine learning of charges and long-range interactions from energies and forces
Source: Nat Commun. 2025 Oct 1;16:8763. doi: 10.1038/s41467-025-63852-x (PMC12488997; doi:10.1038/s41467-025-63852-x)
Supplement: Supplementary file 1 — Supplementary Information [file 41467_2025_63852_MOESM1_ESM.pdf]

Supplementary Information

Machine learning of charges and long-range interactions from energies and forces

Daniel S. King[1,*], **Dongjin Kim**[2,*], **Peichen Zhong**[1,*], **Bingqing Cheng**[1,2,3,†]

[1]Bakar Institute of Digital Materials for the Planet, UC Berkeley, California 94720, United States

[2]Department of Chemistry, UC Berkeley, California 94720, United States

[3]The Institute of Science and Technology Austria, Am Campus 1, 3400 Klosterneuburg, Austria

[*]These authors contributed equally.

E-mail: bingqingcheng@berkeley.edu

S1. LONG-RANGE INTERACTIONS FOR ASYMPTOTIC DECAYING POTENTIALS

One choice for the convergence function is [1]

$$\varphi(r) = \frac{\Gamma(\frac{p}{2}, r^2/2\sigma^2)}{\Gamma(p/2)} = \frac{1}{\Gamma(\frac{p}{2})}\int_{r^2/2\sigma^2}^{\infty} t^{\frac{p}{2}-1}e^{-t}dt, \tag{1}$$

where $\Gamma(m)$ and $\Gamma(m,x)$ are the gamma function and the incomplete gamma function, respectively.

The short-range part of E_p can easily be evaluated in the real space, and the long-range part can be computed in the reciprocal space. The three-dimensional Fourier transform of

$$\mathrm{FT}_3\left[\int_V d\mathbf{r}\sum_{i=1}^{N} q_i\delta(\mathbf{r}_i-\mathbf{r})\int_V d\mathbf{r}'\sum_{j=1}^{N} q_j\delta(\mathbf{r}_j-\mathbf{r}')\right] = |S(\mathbf{k})|^2 \tag{2}$$

and

$$\mathrm{FT}_3\left[\frac{1-\varphi(r)}{r^p}\right] = 2^{3-p}\pi^{3/2}k^{p-3}\Gamma\left(-\frac{p}{2}+\frac{3}{2},\frac{\sigma^2k^2}{2}\right)/\Gamma\left(\frac{p}{2}\right).$$

Using Parseval's theorem,

$$E_p^{\mathrm{lr}} = \frac{2^{2-p}}{V}\frac{\pi^{3/2}}{\Gamma\left(\frac{p}{2}\right)}\sum_{k>0}k^{p-3}\Gamma\left(-\frac{p}{2}+\frac{3}{2},\frac{\sigma^2k^2}{2}\right)|S(\mathbf{k})|^2 \tag{3}$$

where a factor of 2 is absorbed from double counting pairwise interactions. For $p=1$, as $\Gamma(1/2)=\sqrt{\pi}$ and $\Gamma(1,x)=\exp(-x)$,

$$E_1^{\mathrm{lr}} = \frac{2\pi}{V}\sum_{k>0}\frac{1}{k^2}e^{-\sigma^2k^2/2}|S(\mathbf{k})|^2. \tag{4}$$

For $p=6$,

$$E_6^{\mathrm{lr}} = \left(\frac{\pi^{3/2}}{24V}\right)\sum_{k>0}k^3\left[\pi^{1/2}\mathrm{erfc}(b)+\left(\frac{1}{2b^3}-\frac{1}{b}\right)\times\exp(-b^2)\right]|S(\mathbf{k})|^2, \tag{5}$$

where $b^2=\sigma^2k^2/2$, and erfc denotes the complementary error function.

[1] D. E. Williams, Acta Crystallographica Section A: Crystal Physics, Diffraction, Theoretical and General Crystallography **27**, 452 (1971).
