# Supplementary material for: Machine learning of charges and long-range interactions from energies and forces
Source: Nat Commun. 2025 Oct 1;16:8763. doi: 10.1038/s41467-025-63852-x (PMC12488997; doi:10.1038/s41467-025-63852-x)
Supplement: Supplementary file 3 — Transparent Peer Review file [file 41467_2025_63852_MOESM3_ESM.pdf]

nature portfolio

Peer Review File

Machine learning of charges and long-range interactions from energies and forces

Corresponding Author: Professor Bingqing Cheng

This file contains all reviewer reports in order by version, followed by all author rebuttals in order by version.

Version 0:

Reviewer comments:

Reviewer #1

(Remarks to the Author)
The authors report extensions of their recently published Latent Ewald Summation (LES) method (arXiv:2408.15165) that can be used to capture long-range interactions in machine learning interatomic potentials. I very much like that the authors tested their method on numerous examples which are mostly different than in their previous paper. In addition, the manuscript is well-written and technically sound. Nevertheless, there are severe issues with the so-called method extensions, as I elaborate below. In short, in reality, LES doesn't learn physical partial charges but a quantity correlated with partial charges, as authors admit themselves in the text, and evidenced by large deviations found with respect to DFT charge partitioning schemes (Figures 4b, Figure 7). Other extensions are problematic (encoding charge states) or not tested in the paper (imposing charge neutrality). I do not see sufficient novelty in these "extensions" to merit a publication in Nature Communications and suggest redirecting the manuscript to another journal.

1. as the authors admit, there is no theoretical background that would support the claim that the hidden variable q corresponds to actual atomic charge. On the other hand, given that electrostatic interactions are a large, if not the largest, contributor to long-range interactions, it is obvious that this hidden variable will be highly correlated with the partial charge. What is the practical importance of this "extension"? Methods that explicitly learn the partial charges can use this to directly simulate the system under an external electric field, measure the charge transfer, etc. Can you do the same? I don't think so because your MAEs for charges are large (Figures 4b, Figure 7), i.e., much larger, I think, than the differences between different charge partitioning schemes.

2. authors should report the charge errors for all tested systems, i.e., for the Polar dipeptides, please report MAE and R^2 (Figure 4b). The same goes for the test cases of Ko et al. (Table I)

3. charge MAE of LES should be contrasted against the errors of different charge partitioning schemes.

4. claims such as "the ability to learn physical partial charges" in the abstract and "corresponds to the partial charges on atoms" in the conclusion are misleading. As the authors state themselves, they learn a quantity related to the partial charge that should be understood as response charges or coarse-grained approximation of the net electrostatic effect of electron density polarization. And even these claims are not supported by any theory.

5. concatenating local features with the total charge could be problematic in some cases. For example, what would LES predict for the system that includes both Ag_3^+ and Ag_3^- configurations? I don't think you will be able to distinguish the two states because the system's total charge would be zero in this case.

6. the author should comment on the computational overhead of modeling long-range interactions. This analysis is presented in the previous paper, but for completeness, I think it should also be addressed in this paper (e.g., a paragraph in the discussion section).

7. charged molecular dimers example: what does "after removing the mean charge" mean exactly?

(Remarks on code availability)
The code appears to correspond to the previous paper "Cartesian atomic cluster expansion for machine learning interatomic

potentials", npj Computational Materials 2024. and does not include the long-range part or LES method used here. But I could be wrong.

Reviewer #3

(Remarks to the Author)
This is an interesting paper that presents a useful approach to make machine-learning potentials more efficient and realistic, particularly in how they represent long-range electrostatics. The advantages of this method are critically compared against previous approaches and demonstrated on a comprehensive series of test-cases. The variety of the latter provides compelling evidence for the usefulness of the LES approach. However, in my personal opinion, the main strength of the paper lies in the effort to learn from the model, yielding insight into how one could potentially construct better molecular models. It is that latter aspect that I would like to see explored to a greater extent. In general, the paper is suitable for publication, subject to addressing the following comments:

1. In examples A and B, it is shown that the LES method is able to learn the correct charges when fitted against energies and forces arising from a classical model. However, both examples consider only integer ion charges. It is well known that the implicit effects of liquid-phase polarization (more on this below) are better captured by scaled charge ion models (see, e.g. ref [3]). Therefore, I suggest that the authors repeat example B (or A, in fact) using non-integer charges (e.g. ±0.75) that better represent the effects of a polarizing medium.

2. In example B, it would be interesting to decouple the analysis on the bulk system from the interfacial system – i.e. perform them separately. Are the same charges obtained in both? If so, is the learning process “easier” in bulk or at the interface? Are there any anisotropies in ion charges in the interfacial system? It has been hypothesised that effective charges may need to be different from bulk in the close vicinity of an interface, in order to reflect the different polarization environment, so it would be interesting to see if LES is sensitive to such variations.

3. Again in example B, the system size seems quite small to me (64 water molecules), so perhaps it should be demonstrated that finite-size effects are negligible.

4. In example C, the authors state that charges were close to ±1 “after removing the mean charge”. What is meant by this? And what are the implications? It might be worthwhile to compare the LES charges against MBIS, like was done for example D. Are the same trends observed in small charged dimers?

5. In example F, the LES profiles (Figure 8b) also show the presence of a small peak of Cl ions near the interface, whereas that is absent with the SR model. It would be interesting to comment on whether this feature also appears in classical models of electrolyte solutions, i.e. “full-charge”, “partial-charge” and polarizable ion models. This might yield valuable insight into the discussion of which level of polarization is actually needed to accurately describe interfaces of electrolyte solutions.

6. The interpretation of LES charges as “effective charges” that capture the effects of electronic polarization is certainly interesting, but in my opinion, it is insufficiently supported. I think there is a test case that is missing here, which is application to a bulk liquid (e.g. water) simulation using AIMD energies and forces. It has been shown that the concept of scaled charges also applies to non-ionic liquids (see, e.g. doi.org/10.1063/5.0236899 and references therein), so it would be interesting to see if LES is able to “learn” such scaled charges from ab initio data on a liquid, and what is the molecular dipole moment that emerges from such calculations. At the moment, methods to calculate the molecular dipole moment in condensed phases are quite time consuming, so if LES is able to achieve this, it would be quite significant.

7. In connection with the above point, it is clear that LES is able to describe the potential energy surface of molecular systems by learning from energies and forces. However, properties that depend on the dipole moment surface (most notably the dielectric constant) are much harder to predict. In fact, it has been argued (again, see reference above) that non-polarizable models cannot reproduce both surfaces at the same time. I believe that by applying LES to bulk water, the authors could shed some new light into this controversy.

Minor corrections:
- The minimal-basis iterative stockholder method is more commonly referred to using the acronym “MBIS”, so I would suggest that the authors use this instead of “MBI”.
- It would be useful to show the diagonal (i.e. parity line) in Figure 4b.
- Perhaps it would be useful to symmetrize the profiles in Figure 8b to reduce noise – i.e. split the box in the middle and average both sides of the profile together.
- In the second paragraph of the discussion, the authors should also mention charges that are fit to the electrostatic potential (e.g. RESP or CHelpG).

Miguel Jorge, 19/03/2025

(Remarks on code availability)

Reviewer #4

(Remarks to the Author)
Review of "Learning charges and long-range interactions from energies and forces"
This paper presents an impressive and comprehensive framework for learning physical partial charges and accurately modeling long-range electrostatics using the Latent Ewald Summation (LES) method. The authors demonstrate that LES effectively predicts partial charges, dipole and quadrupole moments, and achieves superior accuracy compared to existing approaches that explicitly learn charges. The combination of LES with Cartesian atomic cluster expansion (CACE) further improves the modeling of complex systems with varying charge states and intricate long-range interactions.
I believe this paper is excellent and essentially publishable as is. I particularly admire how the authors demonstrate the ability to learn charges by fitting only to forces, which I consider a significant and impactful result. The extensive amount of work and validation conducted makes this study highly credible and robust. While I believe the paper is essentially ready for publication, I have a few minor comments that the authors may wish to consider:
Message Passing Layers and Long-Range Forces:
Some researchers believe that by stacking multiple layers of message passing, it may be possible to learn long-range forces effectively. It would be interesting to see this tested or for the authors to include a discussion of whether this strategy has been explored in the literature beyond simply extending the cutoff radius.
Alternative Approach by Clementi:
The Clementi group's recent work presents an alternative approach to modeling long-range interactions (see Ref. 10.48550/arXiv.2502.13797). It may be valuable for the authors to briefly discuss the relative advantages and disadvantages of their method compared to Clementi's approach.
Computational Cost:
Including a comment on the additional computational cost associated with LES would be valuable for researchers considering implementing this method in practice.
Table 1:
Should Table 1 include results for the CACE-SR model for completeness? This could help illustrate the relative performance gains from incorporating LES.
Debye-Hückel Screening Length:
Would it be possible to reproduce the Debye-Hückel screening length as a function of concentration? Such a demonstration would be remarkable and highly useful for validating the method's handling of electrolyte screening effects.
Controlling Charge States:
Given the challenges in modeling differing charge states, would it be possible to guide or steer controllable charge states by fixing a particular charge state in the model? This may provide additional flexibility and robustness in LES applications.
Feature Interpretation for Higher-Dimensional q:
The meaning of the features when q is used with dimensionalities higher than one is not extensively discussed. Are there any distinct advantages to using higher-dimensional representations, and what insights can be drawn from this choice?

(Remarks on code availability)
This is the code for the CACE MLIP architecture, which is different from the work presented in this paper.

Version 1:

Reviewer comments:

Reviewer #1

(Remarks to the Author)
The authors have addressed all my comments.

(Remarks on code availability)

Reviewer #5

(Remarks to the Author)
Please note that I was not involved in the first round of review, but I have been asked to look at the reviewer reports (specifically Reviewer #3) and the authors' responses, and advise as to whether I feel that the authors have appropriately addressed those concerns.

Overall, I found this to be an important addition to the field of machine learning potentials. The underlying observation that long-ranged effects can be incorporated without directly learning the (non-physical) partial charges is important, and the methods are demonstrated with low energy and force errors across a range of challenging systems.

In my opinion the authors have responded to the reviewer comments to my satisfaction, and the manuscript is suitable for publication.

Some specific comments on the main points that were brought up in round 1:
- I fully agree that perfect agreement with non-physical charge assignment schemes such as MBIS is not expected here. The

authors have fully demonstrated the differences that can be observed depending on the choice of assignment method in Fig 11, and shown good agreement between quantities computed using their methods and dipole/quadrupole moments that can be computed from QM.
- I agree with the reviewers that investigating the molecular dipole moment and its response to electric fields in a liquid would be of particular importance, but also agree with the authors that there is already a lot covered in this manuscript, and so a follow-up publication seems like a good solution.

Some small suggestions for the authors to consider:
- I'd be interested in a sentence on their thoughts as to whether a transferable version of this method is possible in future. Transferable organic models like ANI and MACE-OFF are seeing a lot of use, and being able to supplement these with accurate long-ranged effects would be of great value.
- Note on page 9, 'titanian' should be 'titanium'

(Remarks on code availability)

Open Access This Peer Review File is licensed under a Creative Commons Attribution 4.0 International License, which permits use, sharing, adaptation, distribution and reproduction in any medium or format, as long as you give appropriate credit to the original author(s) and the source, provide a link to the Creative Commons license, and indicate if changes were made.
In cases where reviewers are anonymous, credit should be given to 'Anonymous Referee' and the source.
The images or other third party material in this Peer Review File are included in the article's Creative Commons license, unless indicated otherwise in a credit line to the material. If material is not included in the article's Creative Commons license and your intended use is not permitted by statutory regulation or exceeds the permitted use, you will need to obtain permission directly from the copyright holder.
To view a copy of this license, visit https://creativecommons.org/licenses/by/4.0/

We thank the referees for their examination of our manuscript. The referees' comments helped to further clarify the advancement in the method and strengthened the demonstrations of our method.

In what follows, we address each of the points raised by the referees. The revision of the manuscript in response to the referees' comments is highlighted in blue in the main text.

Reviewer #1 (Remarks to the Author):

The authors report extensions of their recently published Latent Ewald Summation (LES) method (arXiv:2408.15165) that can be used to capture long-range interactions in machine learning interatomic potentials. I very much like that the authors tested their method on numerous examples which are mostly different than in their previous paper. In addition, the manuscript is well-written and technically sound. Nevertheless, there are severe issues with the so-called method extensions, as I elaborate below. In short, in reality, LES doesn't learn physical partial charges but a quantity correlated with partial charges, as authors admit themselves in the text, and evidenced by large deviations found with respect to DFT charge partitioning schemes (Figures 4b, Figure 7). Other extensions are problematic (encoding charge states) or not tested in the paper (imposing charge neutrality). I do not see sufficient novelty in these "extensions" to merit a publication in Nature Communications and suggest redirecting the manuscript to another journal.

Authors:
We thank the referee for examining our work and for their appreciation of the comprehensive benchmarks in the paper. The referee raised concerns about the method extensions. In the revised paper, we clarify and provide more demonstrations of these extensions:

- We clarify the meaning of "physical charges" in the DFT context, as the different partitioning strategies yield different values. We now provide clear evidence to show that the difference between different DFT partial charge definitions is on par with the difference between the predicted LES and MBIS partial charges.
- Importantly, we now added new evidence that we are able to predict the Born effective charges (BECs) in the dipeptide example. Unlike DFT partial charges such as MBIS, Mulliken charges, and Hirshfeld charges, BECs are unambiguous and experimentally measurable. Detailed responses and explanations are provided below.
- The charge neutrality scheme was not needed in the examples that we tested and was not required due to the tin-foil boundary condition used in the Ewald summation. We have removed it from the main text and put it in the Methods.
- We now explicitly discuss the limitation of encoding charge states. The detailed response and edits are provided below.
- We removed the statement "Extending LES, we incorporate the ability to learn physical partial charges, encode charge states, and the option to impose charge neutrality constraints." in the abstract to reflect that the fact that the main purpose of the paper is not these method extension but to demonstrate the capability of the LES method.

Besides strengthening the methodological aspects of the paper, we have edited the paper to highlight that the main aim is to demonstrate and benchmark the LES method in diverse and challenging contexts. The LES method achieved superior accuracy compared with other long-range machine learning interatomic potentials (MLIPs) that explicitly train on DFT partial charges. Ihe Au2-MgO(001) example, CACE-LR is able to reproduce the DFT energy difference between the dope and undoped substrates, with accuracy far better than any other methods (see Table II, reproduced below).

	DFT	2G-HDNNP	CACE-SR	4G-HDNNP	SevenNet 3-layers	SevenNet 4-layers	CACE-LR
Doped	-66.9	375	431	-41	141	8	-70.6
Undoped	934.8	375	431	975	721	898	931.3

Table II. Energy difference ($E_{\text{wetting}} - E_{\text{nonwetting}}$) in meV between the wetting and nonwetting configurations for doped and undoped substrates. The SevenNet results are from Ref. [48].

In our view, besides the method extensions, the main value of the paper lies in three aspects: 1) we prove that it is neither necessary nor advantageous to explicitly learn from charges. Such insight massively facilitates the construction and usage of long-range MLIPs. 2) We show that the LES method is a very accurate and versatile long-range MLIP method. 3) The method reproduce physical observables including dipoles/quadrupoles and BECs.

1. as the authors admit, there is no theoretical background that would support the claim that the hidden variable q corresponds to actual atomic charge. On the other hand, given that electrostatic interactions are a large, if not the largest, contributor to long-range interactions, it is obvious that this hidden variable will be highly correlated with the partial charge. What is the practical importance of this "extension"? Methods that explicitly learn the partial charges can use this to directly simulate the system under an external electric field, measure the charge transfer, etc. Can you do the same? I don't think so because your MAEs for charges are large (Figures 4b, Figure 7), i.e., much larger, I think, than the differences between different charge partitioning schemes.

Authors:
We thank the referee for this comment. The referee questioned 1) the accuracy of the LES charges compared to the DFT partial charges and 2) the utility of the LES partial charges

We first respond to the question regarding the accuracy of the LES charges with respect to DFT, specifically in the context of the dipeptides (Fig. 4). We want to emphasize that the disagreement between the LES charges and a specific DFT charge scheme (e.g., MBIS, Mulliken charges, and Hirshfeld charges) are not larger compared to the difference between the different DFT charge partitioning schemes. We have calculated Mulliken and Hirshfeld partial charges for the set of dipeptides in PySCF. A pairplot of the different partial charges can be found below. As is seen, the differences between the LES charge and MBIS, Mulliken or Hirshfeld charges are not larger than the difference between the three partial charge schemes.

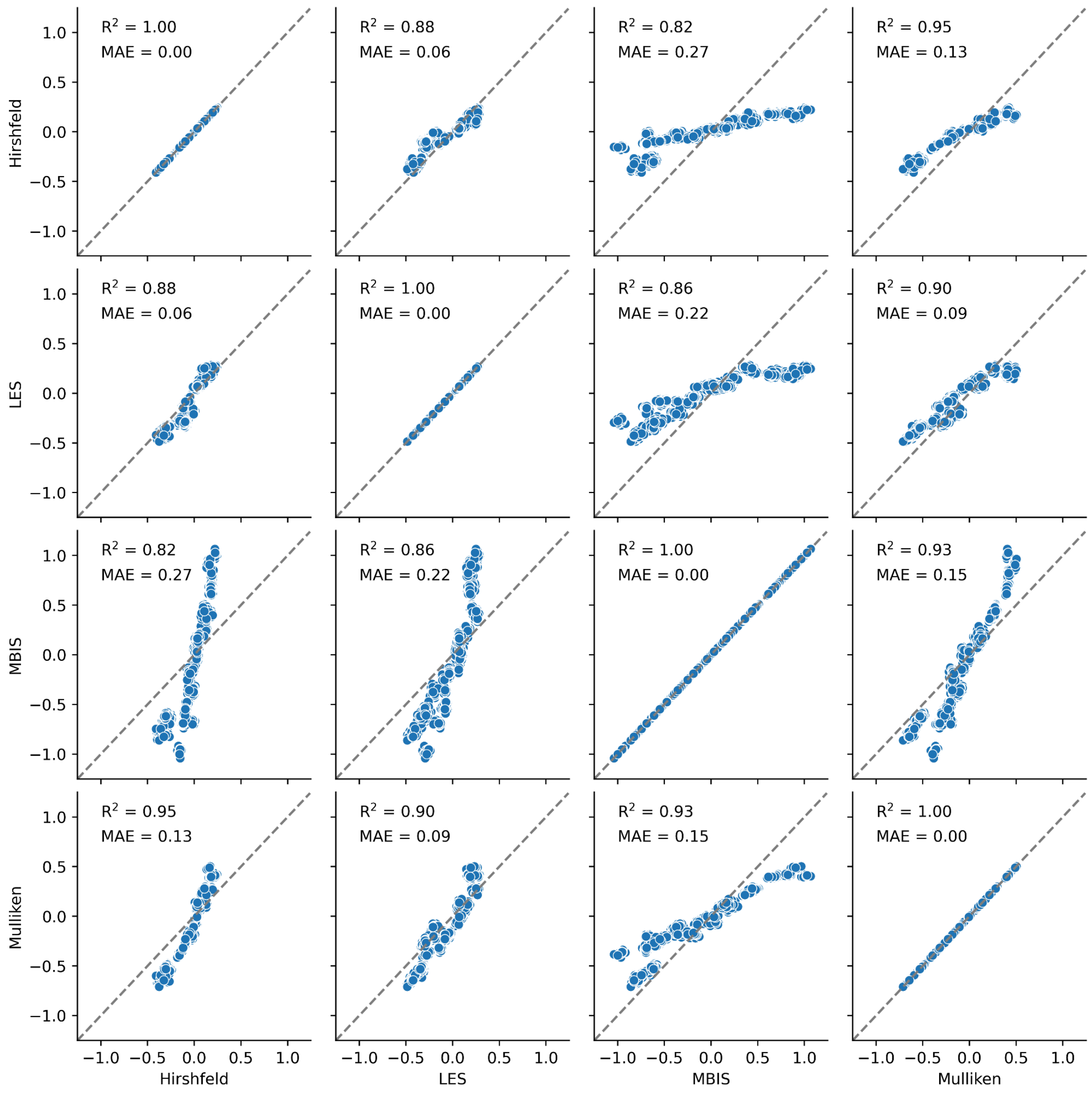

Despite such differences, all the partial charges shown above can more-or-less reproduce the molecular dipoles and quadrupoles (i.e., physical observables) with equal accuracy. We now show this in Fig.10 and reproduce the plot below.

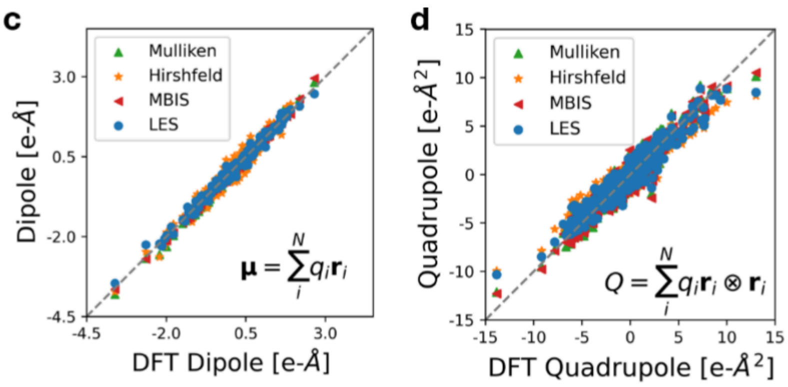

These observations validate our thesis that atomic partial charges are useful only insofar as they can reproduce physical observables like energies, forces, densities, dipoles, quadrupoles, etc. As there is no rigorous definition of DFT partial charges, the comparison between the LES charges and any definition of the DFT charges only serve as an intuitive and illustrative measure and not as a quantitative measure of the physicality of the LES charges. Instead, the quantitative physicality of the LES charges should be inferred from their ability to predict the physical observables such as the dipole and quadrupole moments. In Fig. 4c and 4d, we show that the dipole and quadrupole moments are successfully reproduced by the LES charges. Moreover, in the revised manuscript, we have now calculated and compared another physical observable: the dipole derivative (i.e., the Born effective charge) of the diptides with both DFT and LES (Fig. 4e and 4f and reproduced below). We find that the agreement between DFT and LES for BEC is quite good (figure reproduced below):

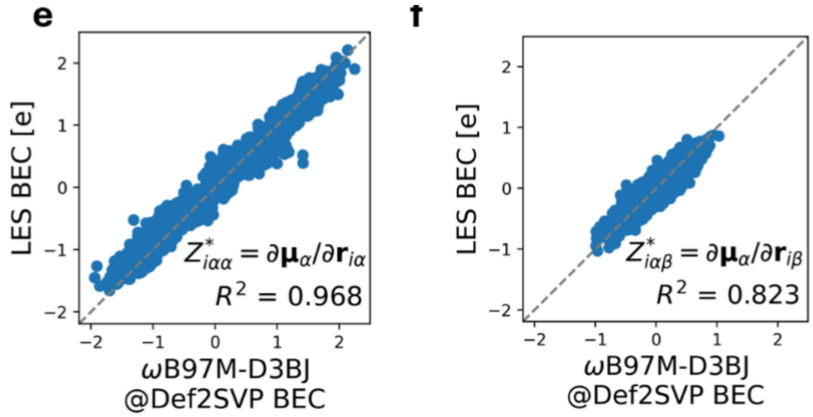

To summarize, in the revised manuscript, we have now clarified and strengthened the argument that the LES charges are indeed physical charges, by 1) more clearly explaining the meaning of the “physical charges” learned by LES, 2) contrasting between different types of DFT partial charges, and 3) showing that the LES charges can reproduce DFT BECs.

We now respond to the second question of the referee regarding the “utility” of the LES charges. We would first like to dig deeper for the referee’s statement that “Methods that explicitly learn the partial charges can use this to directly simulate the system under an external electric field”. Strictly speaking, one should *not* directly use the partial charges (DFT or learned) to simulate

a system under an external electric field. Instead, the quantity to use is the Born effective charge:

$$Z^*_{i\alpha\beta} = \frac{\partial \mu_\alpha}{\partial r_{i\beta}} = \frac{\partial \mathcal{F}_{i\alpha}}{\partial \mathcal{E}^0_\beta}$$

Where mu is the dipole moment, r is the atomic position, E is the external electric field, and F is the response force to the electric field. In models with fixed charges independent of the geometry, the BEC is a scalar equal to the fixed charges; this is not the case for LES and so the BEC must be computed via backpropagation. Given that the LES q can accurately predict BEC, we expect one can use the LES method to simulate systems under external electric fields. Indeed, we have further explored this direction in a new manuscript.

2. authors should report the charge errors for all tested systems, i.e., for the Polar dipeptides, please report MAE and R2 (Figure 4b). The same goes for the test cases of Ko et al. (Table I)

Authors:
As described in Q1 above, we have now reported the charge errors (R2 and MAE) between LES charges and three definitions of the DFT partial charges (MBIS, Mulliken charges, and Hirshfeld charges) for the polar dipeptides. However, the point is that the comparison between LES charges and any definition of the DFT charges is not a quantitative measure of the physicality. The point that we try to make in the paper is that only the ability to reproduce physical observables matters. For the Au2-MgO(001) example in Ko et al, we used physical observables, such as energies, forces, and the surface adsorption curves. Tellingly, although we were not reproducing the Hirshfeld partial charges in the Au2-MgO(001) example (Fig. 7), the accuracy of the CACE-LR is much better than the original study.

3. charge MAE of LES should be contrasted against the errors of different charge partitioning schemes.

Authors:
As described in Q1 above, we have now added these.

4. claims such as ”the ability to learn physical partial charges” in the abstract and ”corresponds to the partial charges on atoms” in the conclusion are misleading. As the authors state themselves, they learn a quantity related to the partial charge that should be understood as response charges or coarse-grained approximation of the net electrostatic effect of electron density polarization. And even these claims are not supported by any theory.

Authors:
We have now modified the abstract and conclusions to explain what we mean by the ability to “learn physical partial charges”.

In the abstract, we now write:

“We show that LES can reproduce the exact atomic charges for classical systems with fixed charges, and it can infer dipole and quadrupole moments, as well as the derivative of the dipole with respect to atomic positions, for quantum mechanical systems. Moreover, LES can achieve better accuracy in energy and force predictions compared to methods that explicitly learn from charges.”

In the conclusion:

“In summary, we thoroughly benchmarked the LES method, a physics-informed approach that learns long-range interactions directly from energies and forces, without requiring explicit charge labels or additional input. We show that LES can achieve better accuracy in energy and force predictions compared to methods that explicitly learn from DFT partial charges.
Moreover, LES is able to learn the true underlying electrostatics: for classical systems with fixed charges, LES can reproduce these exact charges; for quantum mechanical systems, LES can infer dipole, quadrupole moments, and BECs.”

5. concatenating local features with the total charge could be problematic in some cases. For example, what would LES predict for the system that includes both Ag_3^+ and Ag_3^- configurations? I don’t think you will be able to distinguish the two states because the system’s total charge would be zero in this case.

Authors:
Concatenating local features with the total charge of the system has its limitations. For example, if one is dealing with charged systems with varying sizes, the method will break the size consistency. However, for the Ag3-/Ag3+ system the method is appropriate, as the system size is constant.

Just based on the original Ag3-/Ag3+ training set itself, one wouldn’t be able to predict the interactions between Ag clusters. This is because the training set only contains configurations of a single cluster, with all atoms within the cutoff. The multiple cluster scenario is not included in the training set.

We now mention the potential limitations of the global charge embedding scheme in the theory section:
“Note that this global charge embedding scheme can have limitations, for example, when dealing with charged systems with varying sizes.”

6. the author should comment on the computational overhead of modeling long-range interactions. This analysis is presented in the previous paper, but for completeness, I think it should also be addressed in this paper (e.g., a paragraph in the discussion section).

Authors:

We thank the reviewer for the suggestion. We have added a paragraph in the Method section to address this. In summary, we have optimized the LES code, and now the associated computational cost is minimal.

The figure below compares the short- and long-range CACE water models without message passing (T=0) for MD simulations of liquid water on a single NVIDIA L40S GPU with 48GB of memory. In the current work (red curve), we optimized the LR model by first adding the energies using a ‘‘FeatureAdd’ module and then applying autograd to obtain forces. This modification significantly reduced computational cost (~36% improvement compared to the previous implementation that uses two autograd operations to get long-range and short-range forces, green curve). As a result, the computational overhead of including long-range interactions is minimal and the performance becomes comparable to that of the SR model.

All models show favorable scaling. The SR models (T=0) support simulations with up to ~40,000 atoms on a single GPU, while our LR models (T=0) support up to ~13,000 atoms.

We also plan to release the long-range component as a standalone library so that it can be easily integrated with other machine-learning interatomic potentials (MLIPs) in the near future. Additionally, the current CACE model is fully compatible with TorchScript, enabling efficient deployment and further integration in various platforms.

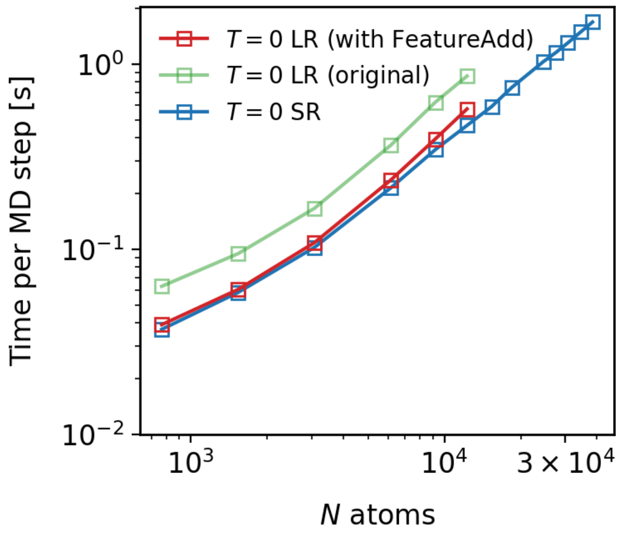

7. charged molecular dimers example: what does ”after removing the mean charge” mean exactly?

Authors:
We took the total charge of the system and computed the mean charge, and then removed this mean charge from each atomic charge. We have revised the text to make this clear:

“after removing the mean charge of each atom $q_i \leftarrow q_i - \sum_i^N q_i/N$”. The reason why the mean charge deviates from zero, and why the atomic charges are not quantitative in this case is due to the tiny training sizes: only 10 dimer configurations are in the training set, and these training structures have the internal coordinates of the molecules frozen and only the distances between the dimers vary. This example aims to illustrate that the LES method can effectively capture the long-range interactions, and provide semi-quantitative predictions of molecular charges, even when the training set has only 10 configurations. We have added these points in the Example B.

Reviewer #1 (Remarks on code availability):

The code appears to correspond to the previous paper "Cartesian atomic cluster expansion for machine learning interatomic potentials", npj Computational Materials 2024. and does not include the long-range part or LES method used here. But I could be wrong.

Authors:
The CACE repository indeed includes the LES method. We have now added a sentence to clarify: The long-range method is implemented as an Ewald module.

Reviewer #3 (Remarks to the Author):

This is an interesting paper that presents a useful approach to make machine-learning potentials more efficient and realistic, particularly in how they represent long-range electrostatics. The advantages of this method are critically compared against previous approaches and demonstrated on a comprehensive series of test-cases. The variety of the latter provides compelling evidence for the usefulness of the LES approach. However, in my personal opinion, the main strength of the paper lies in the effort to learn from the model, yielding insight into how one could potentially construct better molecular models. It is that latter aspect that I would like to see explored to a greater extent. In general, the paper is suitable for publication, subject to addressing the following comments:

Authors:
We thank the referee for the very insightful comments and suggestions. Indeed, the suggestion to more deeply explore the utility of the LES method for learning dipole moment surface for condensed phases coincides with a main idea in our ongoing work titled “Machine learning interatomic potential can infer electrical response”. We attached this new manuscript as material for review. In particular, the suggestion to look at bulk water is an example application in the new manuscript, and the LES method (together with a few theoretical extensions described in the new manuscript) achieved incredible accuracy in predicting the Born effective charges, and even the infrared (IR) spectra of water.

For the revision of the current manuscript, we made the following key changes:

- We clarify what is the meaning of “physical charges” in the DFT context, as the different partitioning strategies yield different values. We now provide clear evidence to show that the difference between different DFT partial charge definitions are on par with the difference between the predicted LES charges and DFT partial charges.
- Importantly, we now added new evidence that we are able to predict the Born effective charges (BEC) in the dipeptide example. Unlike other definitions of DFT charges, BEC are unambiguous and experimentally measurable. The detailed response and explanations are provided below.
- Although a thorough exploration of the charge scaling framework is provided in the main manuscript, in the current manuscript, we added a demonstration and discussion on polarization-environment-dependent charges in the TiO2-NaCl(water) interface example (Example F, Fig.8).

In what follows we respond to the rest of the comments in detail.

1. In examples A and B, it is shown that the LES method is able to learn the correct charges when fitted against energies and forces arising from a classical model. However, both examples consider only integer ion charges. It is well known that the implicit effects of liquid-phase polarization (more on this below) are better captured by scaled charge ion models (see, e.g. ref [3]). Therefore, I suggest that the authors repeat example B (or A, in fact) using non-integer charges (e.g. ±0.75) that better represent the effects of a polarizing medium.

Authors:
We agree that for modeling electrolyte solutions with fixed-charge models, it is more physical to use fractional charges due to the electrical polarization effects. However, examples A and B in the paper aim to show that the LES method is able to learn the charges just from energies and forces. In practice, if we were to repeat the examples using non-integer charges, the Coulomb energy and forces will simply be scaled and the learned energy/forces/charges will be scaled accordingly as well. From the machine learning point of view, such scaling of all outputs would be quite trivial. Moreover, the oxygen and hydrogen atoms in the example B already have non-integer charges (O: -0.8476e H: +0.4238e), and the LES method are able to learn such fractional charges accurately just like the integer charges.

As re-generating the full learning curves (Fig.1 and Fig.2) using scaled charges essentially requires fitting about 50 MLIPs, which would translate to non-negligible amount of effort and GPU hours, we still use the original examples with integer charges for ions (and non-integer charges for O and H). In the main text, we added the following sentence to explain that fractional charges are more suitable for practical simulations:

“It is worth noting, however, scaled charge ion models are typically better at capturing implicit effects of liquid-phase polarization and modeling electrolytes~\cite{kirby2019charge,leontyev2003continuum,jorge2024theoretically}, although this example aims to demonstrate the learning ability of MLIPs rather than to accurately model electrolytes.”

2. In example B, it would be interesting to decouple the analysis on the bulk system from the interfacial system – i.e. perform them separately. Are the same charges obtained in both? If so, is the learning process "easier" in bulk or at the interface? Are there any anisotropies in ion charges in the interfacial system? It has been hypothesised that effective charges may need to be different from bulk in the close vicinity of an interface, in order to reflect the different polarization environment, so it would be interesting to see if LES is sensitive to such variations.

Authors:
This is indeed interesting, and we performed this experiment as suggested by the referee. The figure below shows the learning curves from training only from bulk and only from interfacial structures. Interestingly, the learning efficiency for forces is almost identical for the two sets, but the charges are more difficult to learn from the interfacial systems.

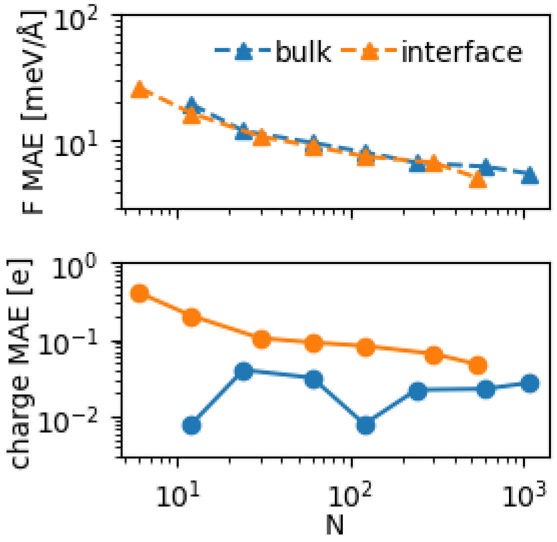

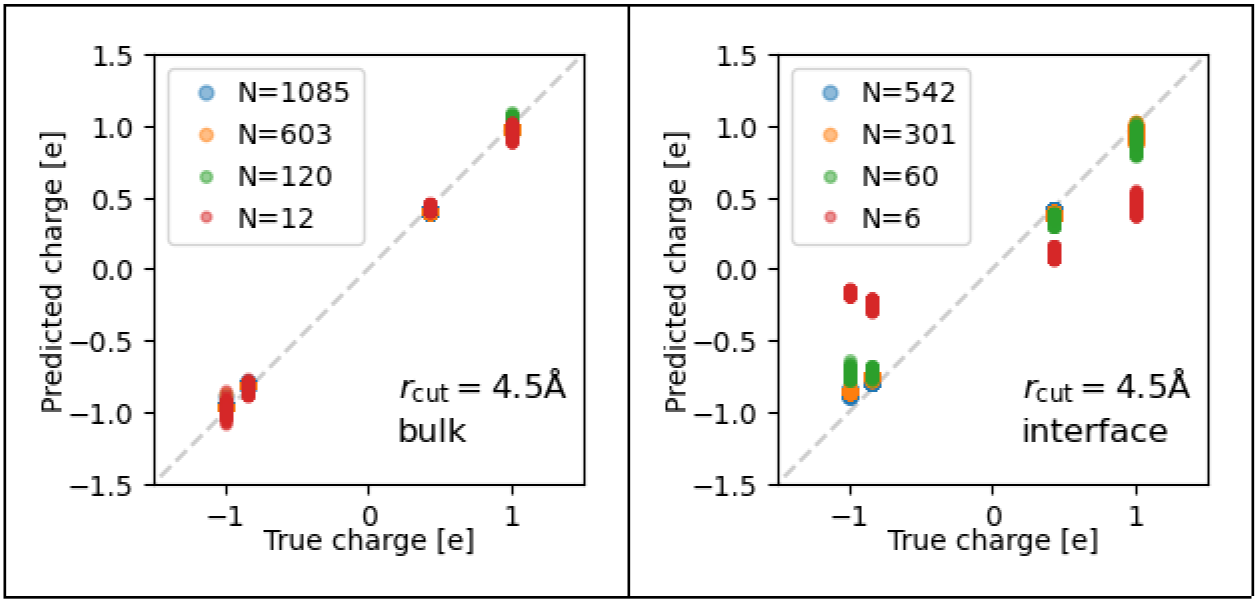

The analysis above is for a classical system with fixed point charges. We also investigate this for an interfacial system (TiO2-NaCl(water) interface (Example F, Fig.8)) described by DFT. We noticed that the learned effective charges are indeed different from the bulk electrolyte to close to the interface, mostly notably for oxygen (red symbols) and titanian (gray symbols). The difference of the effective charges in different medium is related to the different electronic dielectric constants, as we further elucidate in the new manuscript. For interfaces, the electronic dielectric tensor may even be anisotropic. From this example on the interface, one can see that LES is indeed sensitive to the variations of the polarization environments. This is thus an example on the expressiveness of the LES method for complex systems.

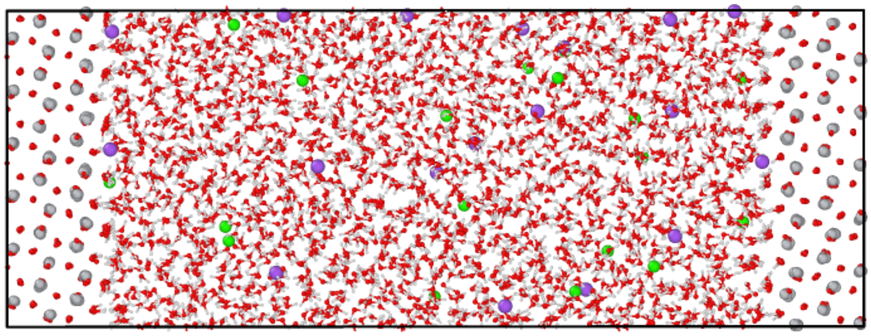

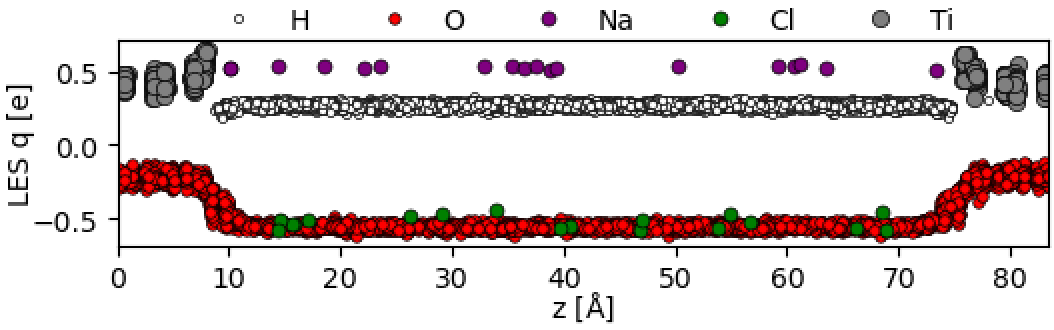

3. Again in example B, the system size seems quite small to me (64 water molecules), so perhaps it should be demonstrated that finite-size effects are negligible.

Authors:
Indeed the system size for the bulk water configurations have 64 water molecules and 0-5 ion pairs, and electrolyte-vapor interfaces configurations have 225 water molecules and 1, 2, or 10 ion pairs. So the system size for the interface configurations are larger.
The system size of 64 water molecules are quite typical for training MLIPs (e.g. https://www.pnas.org/doi/abs/10.1073/pnas.1815117116; https://www.nature.com/articles/s41586-022-05036-x), partly because it would be expensive to generate large training configurations using DFT, and partly because it may be easier to for MLIP to do energy partition (decompose total potential energy into atomic contributions) for smaller systems. It is generally believed that the finite-size-effect is not so important in the training set, as we are only learning from energies and forces. Later on, one should use MLIP to run MD simulations with sufficiently large system sizes.

4. In example C, the authors state that charges were close to ±1 "after removing the mean charge". What is meant by this? And what are the implications? It might be worthwhile to compare the LES charges against MBIS, like was done for example D. Are the same trends observed in small charged dimers?

Authors:
We took the total charge of the system and compute the mean charge, and then removed this mean charge from each atomic charge. We have revised the text to make this clear:
"after removing the mean charge of each atom $q_i \leftarrow q_i - \sum_i^N q_i/N$".

The reason why the mean charge deviates from zero, and why the atomic charges are not quantitative in this case is due to the tiny training sizes: only 10 dimer configurations are in the training set, and these training structures have the internal coordinates of the molecules frozen and only the distances between the dimers vary. We thus did not directly compare the predicted atomic charges to DFT charges, but only the overall molecular charges. This example aims to

illustrate that the LES method can effectively capture the long-range interactions, and provide semi-quantitative predictions of molecular charges, even when the training set has only 10 configurations. We have added these points in the Example B.

5. In example F, the LES profiles (Figure 8b) also show the presence of a small peak of Cl ions near the interface, whereas that is absent with the SR model. It would be interesting to comment on whether this feature also appears in classical models of electrolyte solutions, i.e. “full-charge”, “partial-charge” and polarizable ion models. This might yield valuable insight into the discussion of which level of polarization is actually needed to accurately describe interfaces of electrolyte solutions.

Authors:
This comparison would indeed be insightful. However, we were not able to find any classical MD simulations of TiO2/salt in water electrolyte interfaces. The closest we could find is one paper on TiO2/pure water [https://pubs.acs.org/doi/pdf/10.1021/acs.jpcc.6b12803] using a reactive forcefield.
We suspect the reason is that water can dissociate on the TiO2 rutile(110) surfaces: Surface O_{2c} atoms can accept protons to form the bridging hydroxyl groups, and terminal hydroxyl groups on surface Ti atoms can emerge. In our previous paper that uses MLIP for studying TiO2/water interfaces [https://www.nature.com/articles/s41467-023-41865-8], we estimated the ΔG between dissociated water and molecular water is 5.2 ± 0.6 kJmol^{-1} (with molecular state being more stable) at the optb88-vdw MLP level, 2.6 ± 1.2 kJmol^{-1} at the SCAN MLP level, and 5.1 ± 0.6 kJmol^{-1} at the PBE MLP level. Such free energy differences imply that approximately 10-20% of water molecules dissociate on the rutile(110) surface. This means non-reactive force fields, which the majority of the fixed charge or polarizable classical models are, are not sufficient in describing TiO2/water interfaces.

6. The interpretation of LES charges as “effective charges” that capture the effects of electronic polarization is certainly interesting, but in my opinion, it is insufficiently supported. I think there is a test case that is missing here, which is application to a bulk liquid (e.g. water) simulation using AIMD energies and forces. It has been shown that the concept of scaled charges also applies to non-ionic liquids (see, e.g. doi.org/10.1063/5.0236899 and references therein), so it would be interesting to see if LES is able to “learn” such scaled charges from ab initio data on a liquid, and what is the molecular dipole moment that emerges from such calculations. At the moment, methods to calculate the molecular dipole moment in condensed phases are quite time consuming, so if LES is able to achieve this, it would be quite significant.

Authors:
We thank the referee for this insightful comment. Indeed, we have systematically explored whether LES is able to learn scaled charges from bulk systems in the new manuscript, submitted as materials for referees. We summarize the key conclusions pertinent to the referee’s comment below:

- Essentially, we incorporate the background fast-responding charges into the high frequency relative permittivity ε_∞ (also known as the static or electronic dielectric constant), while explicitly accounting for the Coulomb interactions between the atomic charges. This is similar to the concepts of screened Coulomb interactions and the scaled ion charge in the MDEC model~\cite{leontyev2003continuum,leontyev2011accounting}.
- The LES charges, q_i^{les} are scaled charges, $q_i^{\text{les}} = q_i/\sqrt{\varepsilon_\infty}$, acting both as the sources and the receivers of the electric field. The polarization of a finite system is $\mathbf{P} = \sum_{i=1}^{N} q_i \mathbf{r}_i$, and for a periodic system we use a generalized form: $P_\alpha(k) = \sum_{i=1}^{N} \frac{q_i}{ik} \exp(ikr_{i\alpha})$. One can then obtain Born effective charge tensors (BECs) by taking the derivative of P with respect to atomic positions.
- We believe the accuracy of the LES BEC provides a ``smoking-gun'' validation of the MDEC theory. In addition, our approach is a cleaner way to use the charge scaling framework, as the flexible charges are learned from ab initio data. In comparison, the empirical force fields have the scaled charges and other parameters fitted at the same time to experimental data, which means the errors in describing the electrostatic interactions can be partially canceled by tuning other non-bonded parameters and vice versa, so the resulting charges are less reflective of the true underlying electrostatics. Moreover, the LES framework assigns flexible charges based on local atomic environments. Such environment-dependent charges are more expressive than the fixed charges in empirical force fields. For example, the same LES model is capable of predicting BECs for dramatically different phases of water including isolating ice, superionic ice, and ionic water. It is difficult to imagine a fixed-charge model to match this level of expressiveness.

7. In connection with the above point, it is clear that LES is able to describe the potential energy surface of molecular systems by learning from energies and forces. However, properties that depend on the dipole moment surface (most notably the dielectric constant) are much harder to predict. In fact, it has been argued (again, see reference above) that non-polarizable models cannot reproduce both surfaces at the same time. I believe that by applying LES to bulk water, the authors could shed some new light into this controversy.

Authors:
We thank the referee for this insightful comment. bulk water is an example application in the new manuscript, and there the LES method (together with a few theoretical extensions described in the new manuscript) achieved incredible accuracy in predicting the Born effective charges, and even the infrared (IR) spectra of water.
These new results not only further validate the theoretical foundation of the LES method, but also suggest the tremendous utility of the method for a wide range of complex systems.

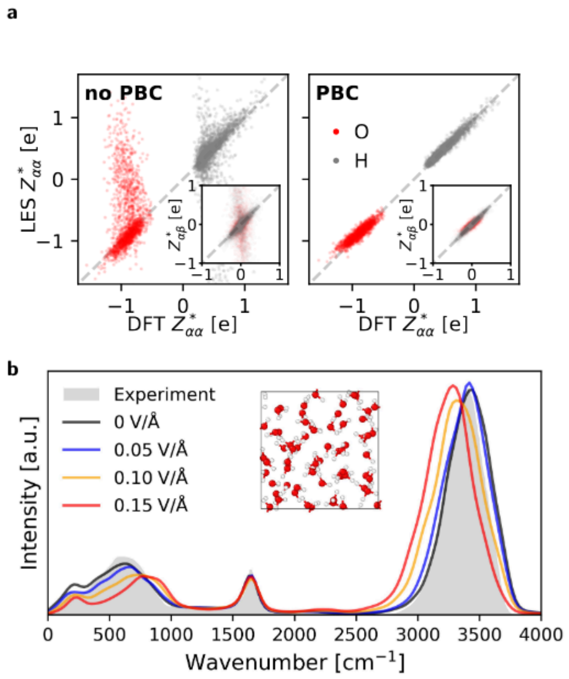

Minor corrections:
- The minimal-basis iterative stockholder method is more commonly referred to using the acronym “MBIS”, so I would suggest that the authors use this instead of “MBI”.

Authors:
All instances of MBI have now been changed to MBIS throughout the paper.

- It would be useful to show the diagonal (i.e. parity line) in Figure 4b.

Authors:
The parity line comparing LES and MBIS charges is now shown; we again emphasize that we expect this agreement to be only qualitative.

- Perhaps it would be useful to symmetrize the profiles in Figure 8b to reduce noise – i.e. split the box in the middle and average both sides of the profile together.

Authors:
We tried this and this is the comparison between the raw density profile and the symmetrized one:

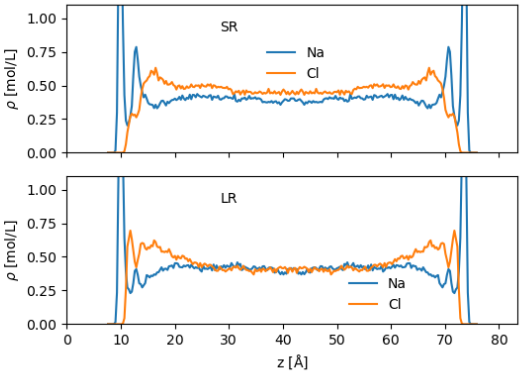

Symmetrized

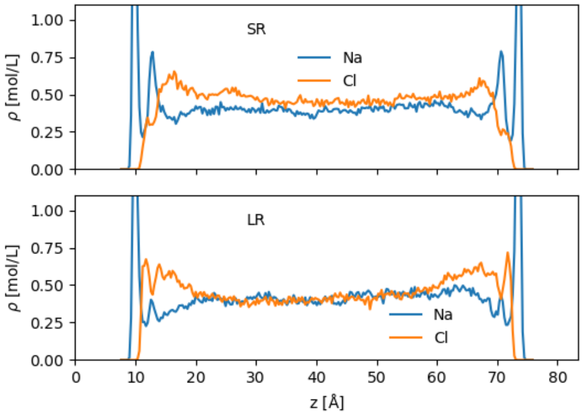

Raw

While the symmetrized version reduced the noise, the difference is not very large and the main features of the density profiles remain the same. We decided to use the raw ones to 1) be more transparent about the statistical uncertainty (the density profile here is not easy to converge and we ran for 5 ns), and 2) allow for a direct comparison with Ref. 47, which made a similar plot for ion density distributions (Supplementary Figure 10) without the symmetrization.

- In the second paragraph of the discussion, the authors should also mention charges that are fit to the electrostatic potential (e.g. RESP or CHelpG).

Authors:
We have added the fitted atomic charges with restraints to the second paragraph of the discussion. (https://pubs.acs.org/doi/abs/10.1021/j100142a004)

Miguel Jorge, 19/03/2025

Reviewer #4 (Remarks to the Author):

Review of "Learning charges and long-range interactions from energies and forces"
This paper presents an impressive and comprehensive framework for learning physical partial charges and accurately modeling long-range electrostatics using the Latent Ewald Summation (LES) method. The authors demonstrate that LES effectively predicts partial charges, dipole and quadrupole moments, and achieves superior accuracy compared to existing approaches that explicitly learn charges. The combination of LES with Cartesian atomic cluster expansion (CACE) further improves the modeling of complex systems with varying charge states and intricate long-range interactions.
I believe this paper is excellent and essentially publishable as is. I particularly admire how the authors demonstrate the ability to learn charges by fitting only to forces, which I consider a

significant and impactful result. The extensive amount of work and validation conducted makes this study highly credible and robust. While I believe the paper is essentially ready for publication, I have a few minor comments that the authors may wish to consider:

Authors:
We thank the referee for reviewing our work and for the positive assessment. We also thank the referee for useful suggestions.

Message Passing Layers and Long-Range Forces:
Some researchers believe that by stacking multiple layers of message passing, it may be possible to learn long-range forces effectively. It would be interesting to see this tested or for the authors to include a discussion of whether this strategy has been explored in the literature beyond simply extending the cutoff radius.

Authors:
This is indeed interesting and (to the best of our knowledge) not thoroughly explored in the literature. One recent paper on this topic [https://pubs.aip.org/aip/jcp/article-abstract/161/24/244102/3327790] showed that that graph neural network interatomic potentials (MACE and SevenNet) can capture non-local electrostatic interactions through many (4-5) message-passing layers. However, 4-5 MP layers are a lot and would cause the potential to be very expensive to evaluate. A typical MACE potential uses 0 or 1 MP layer. Moreover, for the Au2-MgO(001) example, even when using 3 or 4 MP layers, their errors are still much larger than our CACE-LR with 0 MP layers.

	DFT	2G-HDNNP	CACE-SR	4G-HDNNP	SevenNet 3-layers	SevenNet 4-layers	CACE-LR
Doped	-66.9	375	431	-41	141	8	-70.6
Undoped	934.8	375	431	975	721	898	931.3

Table II. Energy difference ($E_{\text{wetting}} - E_{\text{nonwetting}}$) in meV between the wetting and nonwetting configurations for doped and undoped substrates. The SevenNet results are from Ref. [48].

Our previous paper [https://www.nature.com/articles/s41524-025-01577-7] discussed some of this and the observations are:

- For charged molecular dimers, since they live on disconnected graphs, message passing does not help at all.
- For bulk water, simply using message passing to get the long-range interactions leads to the spurious dielectric constant divergence.
- For the water-vapor interface, MLIP with message passing does not give correct dipole screening.

In this work, we also observed that simply adding message passing layers does not give correct dielectrics. In the example of the TiO2-NaCl(water) interface (Example F, Fig.8), we also fitted a short-ranged CACE with 1 message passing layer (MP1 SR). As shown in the figure below, this MP1 SR potential, just like the short-ranged potential without message passing (SR), has the problem of imbalanced ion density in the center of the box.

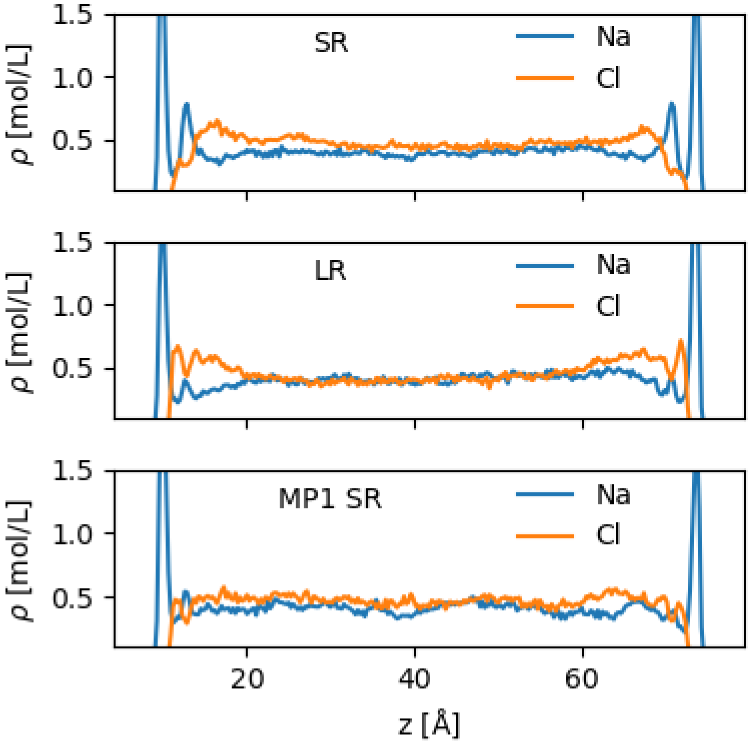

Alternative Approach by Clementi:
The Clementi group's recent work presents an alternative approach to modeling long-range interactions (see Ref. 10.48550/arXiv.2502.13797). It may be valuable for the authors to briefly discuss the relative advantages and disadvantages of their method compared to Clementi's approach.

Authors:
We thank the referee for pointing out this new paper. We have now cited it and discussed it in the introduction:
"RANGE~\cite{clementi2025extending} create global virtual nodes in message-passing graph networks to aggregate and broadcast long-range information."

We think the key advantage of LES is its application in extended systems with periodic boundary conditions, and the interpretability of the learned charges.

Computational Cost:
Including a comment on the additional computational cost associated with LES would be valuable for researchers considering implementing this method in practice.

Authors:

We thank the reviewer for the suggestion. We have added a paragraph in the Method section to address this. In summary, we have optimized the LES code, and now the associated computational cost is minimal.

The figure below compares the short- and long-range CACE water models without message passing (T=0) for MD simulations of liquid water on a single NVIDIA L40S GPU with 48GB of memory. In the current work (red curve), we optimized the LR model by first adding the energies using a ‘‘FeatureAdd’ module and then applying autograd to obtain forces. This modification significantly reduced computational cost (~36% improvement compared to the previous implementation that uses two autograd operations to get long-range and short-range forces, green curve). As a result, the computational overhead of including long-range interactions is minimal and the performance becomes comparable to that of the SR model.

All models show favorable scaling. The SR models (T=0) support simulations with up to ~40,000 atoms on a single GPU, while our LR models (T=0) support up to ~13,000 atoms.

We also plan to release the long-range component as a standalone library so that it can be easily integrated with other machine-learning interatomic potentials (MLIPs) in the near future. Additionally, the current CACE model is fully compatible with TorchScript, enabling efficient deployment and further integration in various platforms.

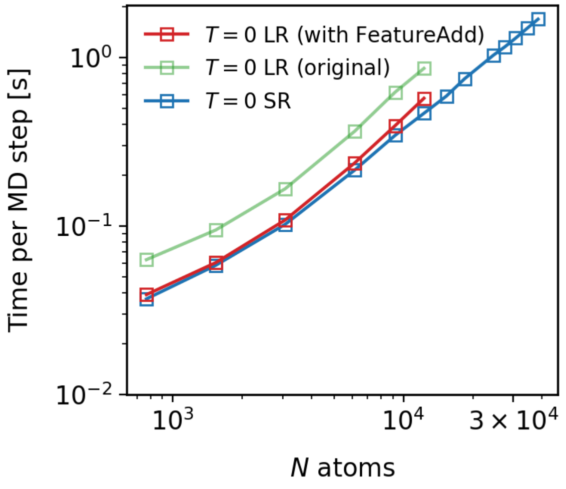

Table 1:
Should Table 1 include results for the CACE-SR model for completeness? This could help illustrate the relative performance gains from incorporating LES.

Authors:
We have now included the CACE-SR results in Table 1:

		ACE	$\chi + \eta$(ACE)	3G-HDNNP	4G-HDNNP	CACE-SR	CACE-LR
$C_{10}H_2/C_{10}H_3^+$	r_{cut}	6 Å	6 Å	4.23 Å	4.23 Å	4.23 Å	4.23 Å
	E	0.76	0.75	2.045	1.194	1.27	0.73
	F	37.22	35.16	231.0	78.00	91.0	36.9
$Ag_3^{+/-}$	r_{cut}	6 Å	6 Å	5.29 Å	5.29 Å	5.29 Å	-
	E	809.62	0.21	320.2	1.323	0.162	-
	F	285.81	23.10	1913	31.69	29.0	-
$Na_{8/9}Cl_8^+$	r_{cut}	6 Å	6 Å	5.29 Å	5.29 Å	5.29 Å	5.29 Å
	E	1.55	0.71	2.042	0.481	1.58	0.21
	F	41.72	12.35	76.67	32.78	48.8	9.78
Au_2-MgO(001)	r_{cut}	6 Å	6 Å	-	4.23 Å	5.5 Å	5.5 Å
	E	2.56	1.63	-	0.219	2.25	0.073
	F	88.70	50.27	-	66.00	59.3	7.91

This shows the improvement from employing LES.

Debye-Hückel Screening Length:
Would it be possible to reproduce the Debye-Hückel screening length as a function of concentration? Such a demonstration would be remarkable and highly useful for validating the method's handling of electrolyte screening effects.

Authors:
This would be a very powerful demonstration. Indeed we have an on-going project that compares long-range and short-range MLIPs in describing Debye-Huckel screening in NaCl/water solutions. The bottleneck is indeed the system size and simulation time convergence. Previous studies (e.g. https://pubs.acs.org/doi/pdf/10.1021/acs.jpcb.9b10542) based on empirical force fields typically use simulation box size of 10nm and simulation time of 50ns. Such settings are expensive using MLIPs.

Controlling Charge States:
Given the challenges in modeling differing charge states, would it be possible to guide or steer controllable charge states by fixing a particular charge state in the model? This may provide additional flexibility and robustness in LES applications.

Authors:
This is a good idea. In principle one can use the global embedding feature to encode the charge state, and fit simultaneously to data with differing charge states. We have not tried this yet, however.

Feature Interpretation for Higher-Dimensional q:
The meaning of the features when q is used with dimensionalities higher than one is not extensively discussed. Are there any distinct advantages to using higher-dimensional representations, and what insights can be drawn from this choice?

Authors:

We have systematically investigated how the dimension of the latent charges (q) affects accuracy and interpretability across various datasets.

The figure below compares potentials with varying dimensions of q (1, 2, 4, 8, 16) on three datasets (Electrolyte solutions, Au2-MgO(001), Polar dipeptides), showing RMSE values for energies (meV/atom) and forces (meV/Å). While a higher-dimensional q can lead to smaller RMSE, the decrease is very modest and there is no significant accuracy advantage.

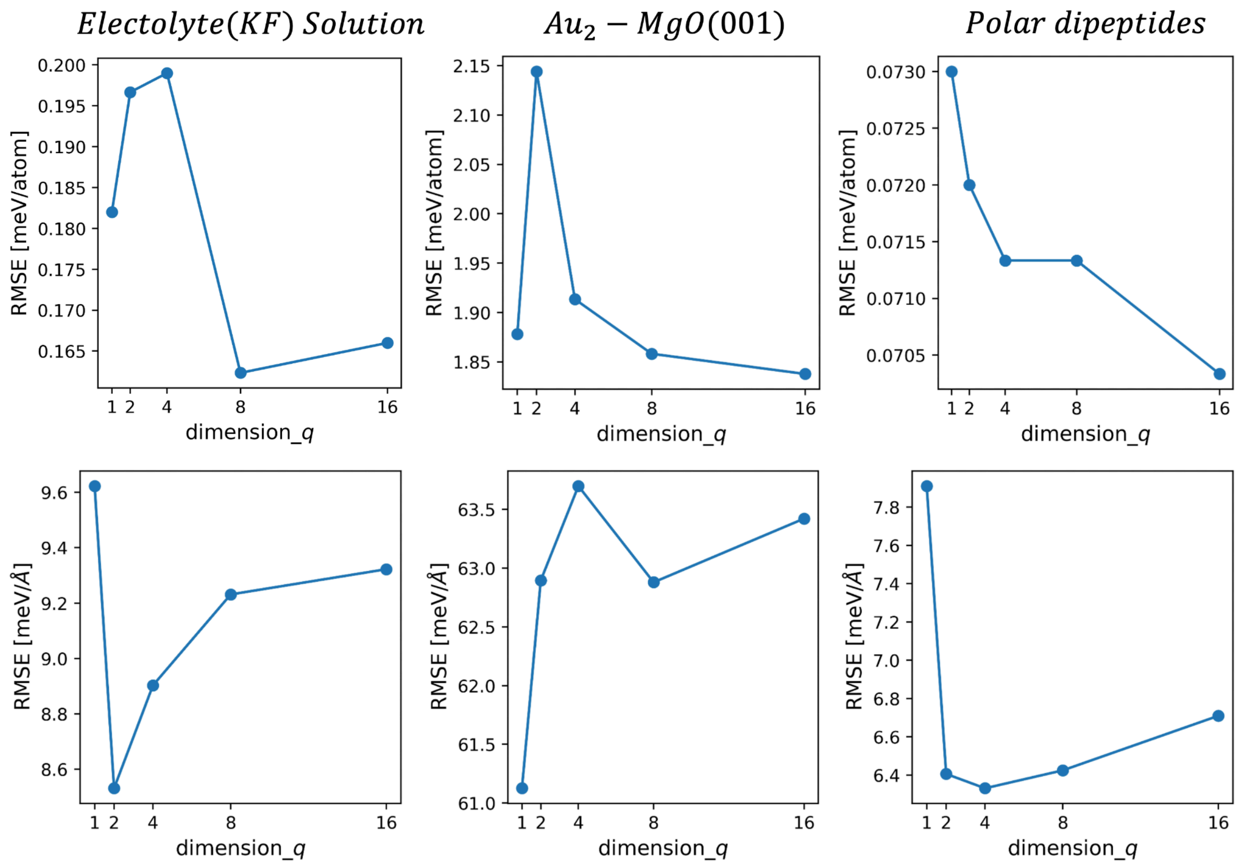

Additionally as shown the figure below, for the electrolyte system(KF aqueous solutions) with ground-truth charges(O: −0.8476e, H: +0.4238e, K: +1e, F: −1e), we compared the LES charges on each channel in various q(1, 2, 4) to the corresponding ground-truth charges. This reveals that despite the presence of multiple dimensions, only a single channel significantly contributes to the total charges. This result shows that, when the ground-truth charge is indeed one-dimensional, the LES method is able to infer the true dimensionality, and the extra channels will not contribute much.

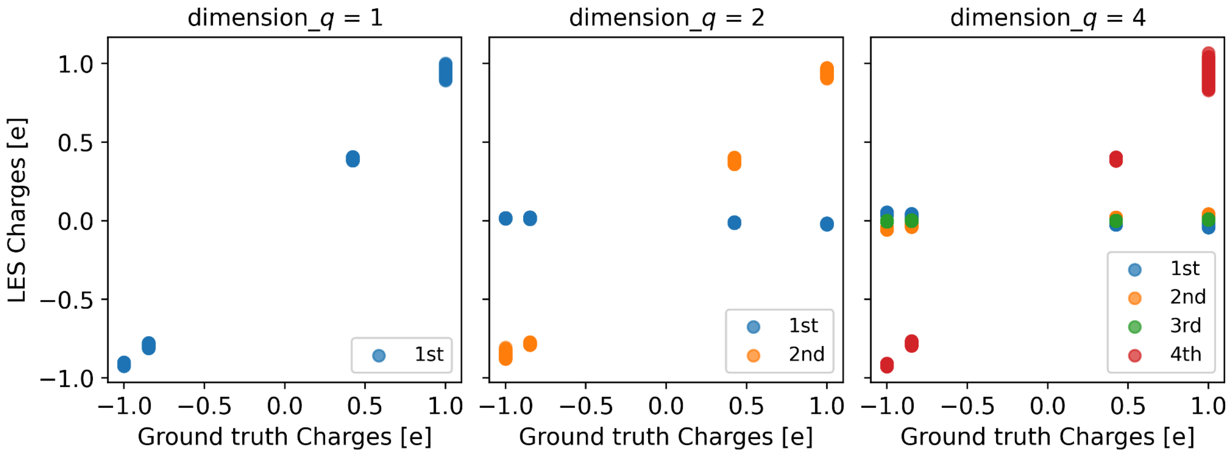

In summary, these analyses thus show two things:

- There is no significant advantage in accuracy in using more charge channels.
- There is no harm either in using more charge channels.

Reviewer #4 (Remarks on code availability):

This is the code for the CACE MLIP architecture, which is different from the work presented in this paper.

Authors:
The CACE repository indeed includes the LES method. We now added a sentence to clarify: The long-range method is implemented as an Ewald module.

Reviewer #5 (Remarks to the Author):

Please note that I was not involved in the first round of review, but I have been asked to look at the reviewer reports (specifically Reviewer #3) and the authors' responses, and advise as to whether I feel that the authors have appropriately addressed those concerns.

Overall, I found this to be an important addition to the field of machine learning potentials. The underlying observation that long-ranged effects can be incorporated without directly learning the (non-physical) partial charges is important, and the methods are demonstrated with low energy and force errors across a range of challenging systems.

In my opinion the authors have responded to the reviewer comments to my satisfaction, and the manuscript is suitable for publication.

Some specific comments on the main points that were brought up in round 1:
- I fully agree that perfect agreement with non-physical charge assignment schemes such as MBIS is not expected here. The authors have fully demonstrated the differences that can be observed depending on the choice of assignment method in Fig 11, and shown good agreement between quantities computed using their methods and dipole/quadrupole moments that can be computed from QM.
- I agree with the reviewers that investigating the molecular dipole moment and its response to electric fields in a liquid would be of particular importance, but also agree with the authors that there is already a lot covered in this manuscript, and so a follow-up publication seems like a good solution.

Authors:
We thank the referee for reviewing our work and the previous response.

Some small suggestions for the authors to consider:
- I'd be interested in a sentence on their thoughts as to whether a transferable version of this method is possible in future. Transferable organic models like ANI and MACE-OFF are seeing a lot of use, and being able to supplement these with accurate long-ranged effects would be of great value.

Authors:
We indeed have long-range transferable machine learning interatomic potentials in the pipeline. We have added a sentence in the discussion section for this:
“In the future, we will incoporate LES into general-purpose MLIPs that are applicable for many systems across the periodic table.”

- Note on page 9, 'titanian' should be 'titanium'

Authors:
Corrected. Thanks.
